# A review of the botany, ethnopharmacology, phytochemistry, analysis method and quality control, processing methods, pharmacological effects, pharmacokinetics and toxicity of codonopsis radix

**DOI:** 10.3389/fphar.2023.1162036

**Published:** 2023-04-06

**Authors:** Jiaojiao Dong, Yexin Na, Ajiao Hou, Shihao Zhang, Huan Yu, Senwang Zheng, Wei Lan, Liu Yang

**Affiliations:** ^1^ Key Laboratory of Basic and Application Research of Beiyao, Ministry of Education, Heilongjiang University of Chinese Medicine, Harbin, China; ^2^ College of Traditional Chinese Medicine, Ministry of Education, Xinjiang Medical University, Xinjiang, China

**Keywords:** codonopsis radix, ethnopharmacology, phytochemistry, analysis method and quality control, processing methods, pharmacological effects

## Abstract

Codonopsis Radix, a traditional Chinese medicine in China, has great medicinal and scientific value. Moreover, it can also be used as a health product in daily diet. This paper reviews the botany, ethnopharmacology, phytochemistry, analysis method and quality control, processing methods, pharmacological effects, pharmacokinetics and toxicity related to Codonopsis Radix. The information of Codonopsis Radix is obtained from scientific databases (such as Baidu Scholar, CNKI, Google Scholar, PubMed, Science Direct, Web of Science, and SciFinder Scholar), Chinese herbal classics, Chinese Pharmacopoeia, PhD and MSc dissertations, and so on. The chemical components mainly include alkaloids, alkynes and polyacetylenes, flavonoids, lignans, steroids, terpenoids, organic acids, volatile oils, saccharides and other components, which have a wide range of neuroprotective effects, protection of gastrointestinal mucosa and anti-ulcer, regulation of body immunity, anti-tumor, endocrine regulation, improvement of hematopoietic function, cardiovascular protection, anti-aging and antioxidant effects. In conclusion, this paper summarizes in depth the shortcomings of the current research on Codonopsis Radix and proposes corresponding solutions. At the same time, this paper provides theoretical support for further research on the biological function and potential clinical efficacy of Codonopsis Radix.

## 1 Introduction

Codonopsis Radix (CR), the Chinese name is Dangshen. According to Chinese Pharmacopoeia (Edition 2020), CR is the dried root of *Codonopsis pilosula* (Franch.) Nannf., *Codonopsis pilosula* Nannf. var.modesta (Nannf.) L. T. Shen and *Codonopsis tangshen* Oliv., and harvested, washed and dried in autumn.

It belongs to the spleen and lung meridians, and has a sweet taste. In addition, it is effective in strengthening the spleen and benefiting the lung, nourishing the blood and promoting the production of body fluid. It is often used to treat deficiency of spleen and lung qi, eat less and feel tired easily, coughing and deficient panting, deficiency of qi and blood, withered face, palpitation and shortness of breath, thirst with fluid, internal heat and thirst.

In recent years, scholars at home and abroad have conducted more in-depth research on CR. Phytochemical research shows that the main chemical components of CR include alkaloids, alkynes and polyacetylenes, flavonoids, lignans, steroids, terpenoids, organic acids, volatile oils, saccharides and other components. In addition, the extract of CR has extensive pharmacological effects, including neuroprotective effects, protection of gastrointestinal mucosa and anti-ulcer, regulation of body immunity, anti-tumor, endocrine regulation, improvement of hematopoietic function, cardiovascular protection, anti-aging and antioxidant effects (Bian et al., 2022). In clinical application, CR is often used in combination with other traditional Chinese medicines (TCM), it can mainly treat anemia, hypotension, digestive disorders, acute plateau reaction (Liu et al., 2019; Wang et al., 2019). Moreover, there are studies and reports indicating that CR has almost no toxic side effects (Feng and Gao, 2012). Furthermore, CR is also widely used in healthcare medicines, health food, healthcare cosmetics and other fields, such as drinks, tea, biscuits, medicated meals, moisturizing cream, *etc.*, (Lei et al., 2022).

CR is a valuable botanical drug that has received a lot of attention for its unique medicinal value and healthcare effects. In this study, we collected and organized the literature on CR from 1982 to the present. Except for duplicates and irrelevant literature, a total of 192 articles related to the botany, traditional applications, phytochemistry, pharmacology, toxicity, analytical methods and quality control, processing methods, and pharmacokinetics of CR were systematically summarized in this paper, to provide references for further development and utilization of CR and to explore possible research directions and new prospects of CR. In conclusion, this review is of great significance for further research and utilization of CR.

## 2 Botany

**FIGURE 1:**
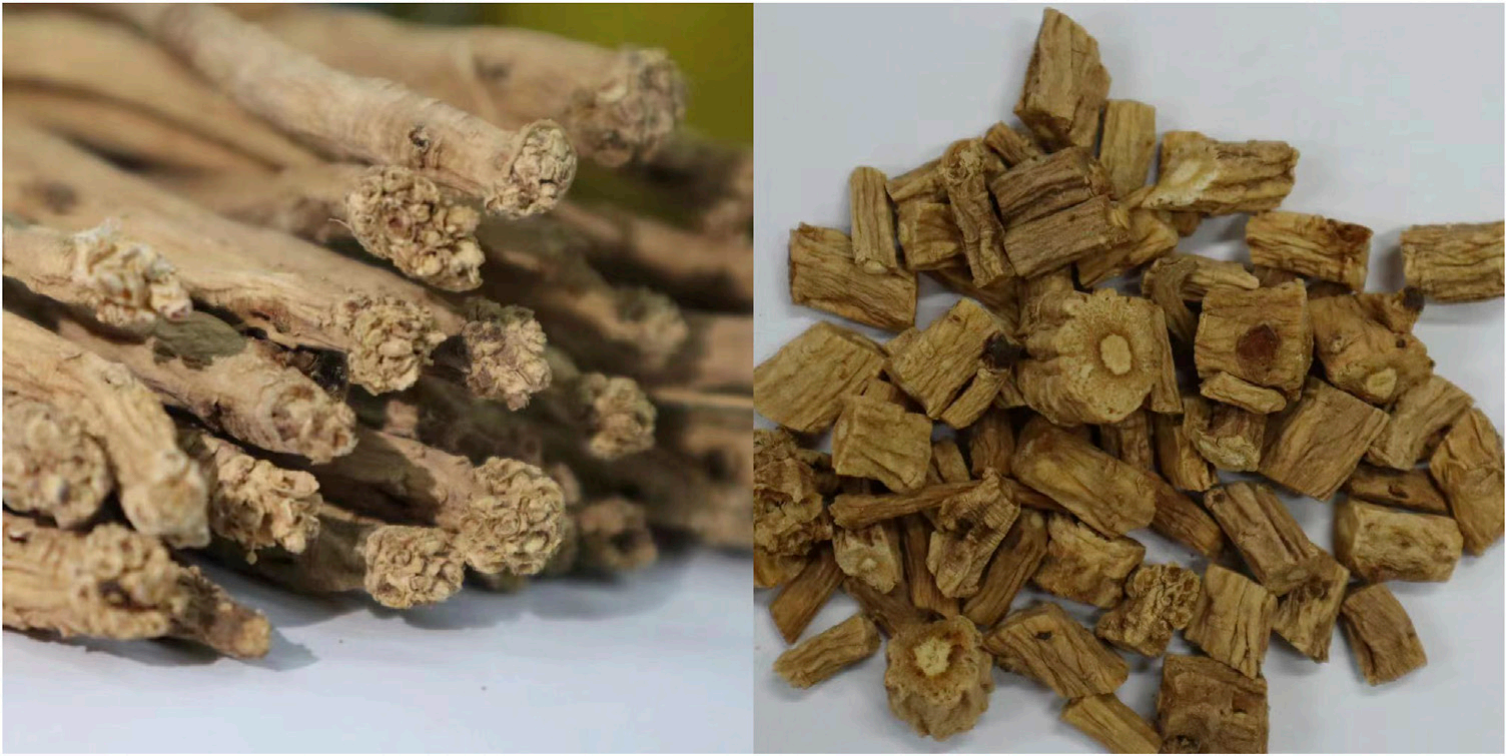
The pictures of CR and its slices.

Codonopsis Radix, a perennial botanical drug of Campanulaceae. The stem is usually very short, and most of them have tumor-like stem marks. The roots are often plump, cylindrical, conical, spindle-shaped, block-shaped, oval, spherical or rosary, and are fleshy or woody. Stems erect or twining, climbing, inclined, ascending or procumbent. Leaves alternate, opposite, clustered or pseudowhorled. Flowers are borne singly at the tips of the main stem and lateral branches, opposite the petiole, and less frequently in the axils of the leaves, sometimes in scape form. CR enjoys a mild and cool climate, is cold-tolerant, and its roots can overwinter in the open ground in the soil. It is born at the mountain forest edge and shrub at an altitude of 1560–3100 m. The pictures of CR and its slices are shown in Figure 1.

There are more than 40 species in the whole genus of *Codonopsis*, distributed in eastern and central Asia, about 39 species in China, 21 species for medicinal purposes and 4 varieties. The Flora of China records that CR is produced in southeastern Tibet, western Sichuan, northwestern Yunnan, eastern Gansu, southern Shaanxi, Ningxia, eastern Qinghai, Henan, Shanxi, Hebei, Inner Mongolia and northeastern China, and is cultivated in large quantities throughout the country. The species of Radix Codonopsis are diverse and widely distributed, and most of them are named after their traits, origin, processing characteristics, *etc.*, the phenomenon of different substance with the same name and different meaning for the same items often occurs (Bian et al., 2022). In addition, some local Codonopsis Radix is better than genuine Codonopsis Radix in certain pharmacological activities. For example, *Codonopsis clematidea* (Schrenk) C.B.Clarke [Campanulaceae; Xinjiang Dangshen] (CCS) polysaccharides can improve SOD activity and reduce MDA production, and its anti-free radical effect is better than that of Lu Dangshen (Xiong et al., 2000). In some areas, *Codonopsis clematidea*, *Codonopsis nervosa* (Chipp) Nannf. [Campanulaceae; Maihua Dangshen]and *Codonopsis lanceolata* (Sieb. et Zucc.) Trautv. [Campanulaceae; Lunye Dangshen] are also often used as medicine. According to “the ConPhyMP statement”, we sorted out the botanical drugs involved in the article (Rivera et al., 2014; Heinrich et al., 2022). Besides, we compiled the geographical distribution of common medicinal Codonopsis (genuine and local customary) and their identification methods, as shown in Table 1. However, the source of CR is complex, and the quality of different producing areas varies greatly, exists the phenomenon of substitute shoddy goods for good cargo. Therefore, it is necessary for us to establish a scientific, systematic and reasonable quality control standard and evaluation system of CR to provide assurance for safe and reasonable clinical use. In addition, the sources and characteristics of the confusions of CR are shown in Supplementary Table S1.

**TABLE 1:** Distribution and identification of common medicinal CR. (Genuine and local products).

Applied range	Latin name	Chinese name	Trade name and distribution area	Pharmacognostic properties
Genuine CR	Codonopsis pilosula (Franch.) Nannf	Dangshen	Lu Dang: Southeast Shanxi Tai Dang; Wutaishan, Shanxi Baitiao Dang: Dingxi and Longxi in Gansu Ban Dang: Hubei Enshi ban qiao	Long cylindrical, 10–35 cm long, 0.4–2 cm in diameter; Texture slightly soft or hard and ductile, flat section; It has special aroma and slightly sweet taste
	Codonopsis pilosula Nannf.var.modesta (Nannf.) L.T.Shen	Suhua Dangshen	Wen Dang: Wen County, Gansu Jing Dang:Nanping County, Sichuan	10–35 cm long and 0.5–2.5 cm in diameter. The surface is yellowish-white or grayish-yellow, with dense ring-shaped horizontal stripes at the lower end of the root, and many cracks in the section
	Codonopsis tangshen Oliv	Chuan Dangshen	Chuan Dang: Northern and eastern Sichuan, northern Guizhou*etc.*	10–45 cm long and 0.5–2 cm in diameter. The surface is grayish yellow to yellowish brown with obvious irregular longitudinal grooves; There are few cracks in the section
Local CR	*Codonopsis clematidea* (Schrenk) C. B. Cl	Xinjiang Dangshen	Xinjiang, western Tibet, India, Pakistan, Afghanistan and Central Asia of the Soviet Union	The roots are usually hypertrophic and spindle-shaped and cylindrical, with a length up to 25–45 cm and a diameter of 1–3 cm. The surface is grayish yellow, with fine ringlike grains near the upper part, while the lower part is sparsely covered with transverse long lenticels
	*Codonopsis foetens* subsp. *nervosa* (Chipp) D. Y. Hong	Maihua Dangshen	Northwest Sichuan, East Tibet (Suoxian, Luwuqi, Qamdo and Jiangda), Southeast Qinghai and South Gansu	Roots often hypertrophic, terete, 15–25 cm long, 1–2 cm in diameter, yellowish-gray on the surface, with a few ringlets near the upper part, and sparsely transversely elongated lenticels at the lower part
	*Codonopsis lanceolata* (Sieb. et Zucc.) Trautv	Lunye Dangshen	Yangru: Northeast China, North China, East China and South-central China	Stem base nearly conical or terete, root usually hypertrophic and spindle-shaped. 10–20 cm long, surface grayish yellow, sparsely ringlike near upper part, and sparsely transversely elongated at lower part
	*Codonopsis thalictrifolia* Wall. var. mollis (Chipp) L. T. Shen	Changhua Dangshen	Southern and central Tibet in China	The stems are mostly erect or ascending, and the flowers are light blue. Roots often hypertrophic, oblong-conical or terete, 15–20 cm long, 0.5–1 cm in diameter, pale yellow on surface
	*Codonopsis canescens* Nannf	Huimao Dangshen	Produced in western Sichuan, eastern Tibet and southern Qinghai	Stems with many fine stalk marks, coarser and longer and erect. Roots often hypertrophic spindle-shaped and less branched, 20–30 cm long, 1–2.5 cm in diameter, pale yellow on surface

## 3 Ethnopharmacology

### 3.1 Traditional uses

CR is a commonly used tonic botanical drug in China, through textual research of ancient books, CR has a long medicinal history, and it has been mixed with ginseng for 1,000 years. The name of Dangshen was first recorded in the Qing Dynasty’s “Baicaojing”, and before that there were similar records in the “Bencao”, but the name of Dangshen was not mentioned. In this section, we have compiled a list of famous writers on CR since it was recorded, to facilitate the better exploitation of Codonopsis Radix.

It is recorded in Ben Jing Feng Yuan (Qing Dynasty, 1617–1700) that the Shang Dang Ren Shen, though not capable of nourishing and warming, has the power of calming and clearing the lung, and it is not as cold as the *Glehnia littoralis* (A.Gray) F. Schmidt ex Miq. [Apiaceae; Glehniae Radix]. According to the New Compilation of Materia Medica (本草从新, AD 1757), it can tonify the stomach and spleen, and eliminate polydipsia. This effect is consistent with the discovery in modern pharmacology that CR protects the gastrointestinal tract and enhances the function of the spleen and stomach. Gang Mu Shi Yi (纲目拾遗, AD 1765) recorded that CR can cure lung deficiency and benefit lung qi. It is recorded in “De Pei Ben Cao” (得配本草, AD 1644–1911) that when Codonopsis pilosula is served and steamed with honey, it can tonify the lungs. According to Ben Cao Zheng Yi (本草正义, AD 1920), CR can nourish the spleen and stomach, moisten the lungs and produce fluid, and the tendons transport qi, which is not far from *Panax ginseng* C. A. Mey. [Araliaceae; Ginseng Radix Et Rhizoma]. Modern pharmacological studies have proved that the functions of CR in enhancing immunity, nourishing stomach and invigorating spleen are consistent with those in ancient books. In addition, we learned from ancient books that the efficacy of CR and Ginseng Radix Et Rhizoma was similar, and it was once used as a substitute for Ginseng Radix Et Rhizoma in clinical application. In general, for chronic asthenia such as spleen and stomach deficiency, lung qi deficiency, and body fatigue and weakness, CR can be used to treat these diseases instead of Radix Ginseng, and the two have been mixed for thousands of years.

### 3.2 Herb pairs applications

Herb pair compatibility is not the stacking of any two drugs, but rather the pairing of drugs according to their properties, with the drugs cooperating with or restricting each other to achieve a better curative effect. It contains the wisdom and clinical experience of past dynasties of TCM physicians, and is a relatively fixed collocation form of two drugs in clinical TCM compatibility.

CR, under the guidance of Chinese traditional medicine theory, is often used in combination with other drugs. The compatibility of CR and *Angelica sinensis* (Oliv.) Diels [Apiaceae; Angelicae Sinensis Radix] can be used for patients with spleen and lung deficiency and can significantly enhance the immune function of the body (Wang et al., 2017a; Wang et al., 2017b). CR is combined with *Atractylodes macrocephala* Koidz. [Asteraceae; Atractylodis Macrocephalae Rhizoma], *Poria cocos (Schw.) Wolf* [Polyporaceae; Poria], and *Glycyrrhiza uralensis* Fisch. ex DC. [Fabaceae; Glycyrrhizae Radix Et Rhizoma] to treat spleen and stomach deficiency, anorexia, loose stool, and fatigue due to qi deficiency (Chen S. et al., 2002). Compatibility with *Astragalus mongholicus* Bunge [Fabaceae; Astragali Radix] can strengthen the effect of invigorating qi and enhancing immunity, and it is the most commonly used tonic traditional Chinese medicine (Zhou and Zhou, 2011; Shen and Jin, 2012). CR is combined with Atractylodis Macrocephalae Rhizoma, Angelicae Sinensis Radix, and *Rehmannia glutinosa* (Gaertn.) DC. [Orobanchaceae; Rehmanniae Radix Praeparata] to invigorate both qi and blood, and is used for treat sallow complexion, palpitation, and dizziness due to blood deficiency (Feng and Zhang, 2016). CR is combined with *Schisandra chinensis* (Turcz.) Baill. [Magnoliaceae; Schisandrae Chinensis Fructus] and *Ophiopogon japonicus* (L. f.) Ker Gawl. [Asparagaceae, Ophiopogonis Radix] for treating lung, kidney, and fluid injury, manifested as asthenia, soreness of waist and spermatorrhea, with the effects of invigorating qi, nourishing yin, and promoting fluid production (Yan et al., 2015).

### 3.3 Prescription applications

CR is a traditional botanical drug commonly used in China and one of the most valuable herbs typically used. The commonly used clinical prescriptions include Shengmaiyin (Dangshen Prescription), Shenqi Baizhu Decoction, Shiquan Dabu Pill and so on. Among them, Shengmaiyin is most widely used in clinical practice and has the effects of benefiting qi, nourishing yin and promoting fluid production. In addition, the common dosage forms of CR involved decoction, pill, tablet, granule, capsule, syrup, *etc.* The auxiliary prescriptions of CR commonly used in clinic summarized from the ancient books of traditional Chinese medicine and China Pharmacopoeia are shown in Table 2.

**TABLE 2:** The prescription composition, dosage form, and efficacy of CR.

NO.	Prescription name	Major component	CR dosage	Formulation	Traditional and clinical uses	Ref
1	Shengmaiyin (Dangshen Prescription)	Codonopsis Radix, Ophiopogonis Radix, Schisandrae Chinensis Fructus	150 g	Decoction	Benefiting qi and restoring pulse, nourishing yin and generating body fluid	Zhong Yao Cheng Fang Zhi Ji, Volume 10
2	Shangdangshengao	Codonopsis Radix, Glehniae Radix (Adenophorae Radix), longan pulp	500 g	Paste	Clearing lung and invigorating primordial qi	De Pei Ben Cao
3	Shenqibaizhutang	Codonopsis Radix, Astragali Radix, Atractylodis Macrocephalae Rhizoma, nutmeg frost, Poria, Dioscoreae Rhizoma, Cimicifugae Rhizoma, Glycyrrhizae Radix Et Rhizoma	10 g	Decoction	Treatment of diarrhea and procreation of proctoptosis due to qi deficiency	Bu Zhi Yi Bi Yao
4	Shenqianweisan	Codonopsis Radix, Astragali Radix, Poria, Glycyrrhizae Radix, Paeoniae Radix Alba	10 g	Decoction	For injuries to spleen and stomach and oral sores caused by the administration of cold medicines	Hou Ke Zi Zhen Ji
5	Liujunzi Wan	Codonopsis Radix, Poria, Atractylodis Macrocephalae Rhizoma, Pinelliae Rhizoma Praeparatum Cum Zingibere Et Alumine, Citri Reticulatae Pericarpium, Glycyrrhizae Radix Et Rhizoma Praeparata Cum Melle	200 g	Pill	Tonifying spleen and qi, eliminating phlegm. Can be used for treating spleen and stomach deficiency, food intake deficiency, qi deficiency and excessive phlegm	Chinese Pharmacopoeia, 2020 edition
6	Shiquan Dabu Wan	Codonopsis Radix, Atractylodis Macrocephalae Rhizoma, Poria, Glycyrrhizae Radix, Angelicae Sinensis Radix, Chuanxiong Rhizoma, Paeoniae Radix Alba, Rehmanniae Radix Praeparata, Astragali Radix, Cinnamomi Cortex	80 g	Pill	For the treatment of Qi and Blood deficiency, pale complexion, shortness of breath and palpitations, dizziness and sweating, body fatigue and weakness, lack of warmth in the limbs, heavy menstruation	Chinese Pharmacopoeia, 2020 edition
7	Bazhen Wan	Codonopsis Radix, Atractylodis Macrocephalae Rhizoma, Poria, Glycyrrhizae Radix, Angelicae Sinensis Radix, Paeoniae Radix Alba, Chuanxiong Rhizoma, Rehmanniae Radix Praeparata	100 g	Pill	Used for deficiency of Qi and blood, yellowing of the face, loss of appetite, weakness of the limbs, excessive menstruation	Chinese Pharmacopoeia, 2020 edition
8	Erkangning Tangjiang	Codonopsis Radix, Atractylodis Macrocephalae Rhizoma, Dioscoreae Rhizoma, Ophiopogonis Radix、 Astragali Radix, Poria Coicis Semen, Polygoni Multiflori Radix Praeparata, Jujubae Fructus, Crataegi Fructus, Hordei Fructus Germinatus, Mori Ramulus	60 g	Syrup	Replenish qi and invigorate spleen, promote digestion and stimulate appetite	Chinese Pharmacopoeia, 2020 edition
9	Shandong Ejiao Gao	Asini Corii Colla, Codonopsis Radix, Atractylodis Macrocephalae Rhizoma, Astragali Radix, Lycii Fructus, Paeoniae Radix Alba, Glycyrrhizae Radix	80 g	paste	It is used for coughing due to deficiency of Qi and Blood, vomiting of blood, women’s leakage and fetal disturbance	Chinese Pharmacopoeia, 2020 edition
10	Xiao’er Fupi Keli	Atractylodis Macrocephalae Rhizoma, Citri Reticulatae Pericarpium, Crataegi Fructus, Codonopsis Radix, Nelumbinis Semen, Poria	48 g	granule	Strengthening the spleen and stomach, helping digestion. Used for children with spleen and stomach deficiency, indigestion, emaciation	Chinese Pharmacopoeia, 2020 edition
11	Xiaochaihu Pian	Bupleuri Radix, Astragali Radix, Glycyrrhizae Radix, Jujubae Fructus, Pinelliae Rhizoma Praeparatum Cum Zingibere Et Alumine, Codonopsis Radix, Zingiberis Rhizoma Recens	167 g	tablet	Relieving exterior syndrome and dissipating heat, soothing liver and harmonizing stomach. Can be used for treating inappetence, vexation, vomit, bitter taste in mouth, and dry throat	Chinese Pharmacopoeia, 2020 edition
12	Niuhuang Jiangya Jiaonang	Saigae Tataricae Cornu, Margarita, Powerdered Buffalo Horn Extract, Bovis Calculus Artifactus, Borneolum Syntheticum, Paeoniae Radix Alba, Codonopsis Radix, Astragali Radix, Cassiae Semen, Chuanxiong Rhizoma, Scutellaria Extract, Nardostachyos Radix Et Rhizoma, Menthae Haplocalycis Herba, Curcumae Radix	Unknown	Capsule	Clearing heart fire and eliminating phlegm, calming liver and tranquilizing mind. Can be use for treating heart fire, headache, insomnia, and dysphoria	Chinese Pharmacopoeia, 2020 edition

### 3.4 Others

As we all know, CR is one of the traditional Chinese medicines with homology of medicine and food. In some parts of China, CR is often used as food in daily diet, such as porridge, soup, stew, bubble wine, tea, *etc.* According to “China Medicated Diet Dictionary”, CR is a commonly used qi-reinforcing drug in medicinal diet for tonifying the middle, replenishing qi, and promoting fluid production, and is often used for the treatment of spleen and stomach deficiency, qi and blood deficiency, fatigue and weakness. In addition, Li Yan and others compiled “Miao Yong Dangshen Zhi Bai Bing”, which selected more than 100 therapeutic prescriptions of CR summarized by physicians of past dynasties. Besides, Codonopsis lanceolata has been listed as edible wild vegetable for a long time in China’s border and North Korea, South Korea, Japan and other regions without any toxic or side effects. In 2019, the National Health and Wellness Commission of China and State Administration for Market Regulationissued the Notice on the pilot work of material management for 9 kinds of substances, such as Codonopsis pilosula, which are traditionally both food and Chinese herbal medicines. Hebei Province, Gansu Province and other places have carried out pilot work of Codonopsis pilosula in combination with local characteristics. In recent years, there are research teams developed Dangshen biscuits, Dangshen Gao, Dangshen Fu, Dangshen nougat, Dangshen Mi-jian, Dangshen-Huang-qi drinks and other products. Therefore, we organized some common recipes of CR and their effects, as shown in Table 3. In addition, studies have also reported the application of CR in beauty and skin care products. Codonopsis oligosaccharides have anti-aging effect and can be used in emollient creams (Zhou, 2021).

**TABLE 3:** Common CR recipes and their effects.

NO.	Medicine and food homology	Effects	Origin
1	Dangshen Zhou	Deficiency of spleen and stomach, tiredness and fatigue, shortness of breath, and weakness of qi and blood after illness	Chang Jian Bing Shi Liao Shi Bu Da Quan
2	Dangshen Baihe ZhufeiTang	Benefiting Qi and nourishing the lung. It is used for shortness of breath, coughing and phlegm, chest tightness and pallor caused by tuberculosis	Ji Bing De Shi Liao Yu Yan Fang
3	Shengui ZhuganTang	Indicated for palpitations, insomnia and yellowing of the face due to deficiency of blood in the heart and liver	Si Chuan Zhong Yao Zhi
4	Shen Zao Tang	Is suitable for anemia patients due to deficiency of both qi and blood	Shi Yao Shen Shu
5	Qi Shen Yang Rou Geng	Can be used for treating qi and blood deficiency, sallow complexion, dizziness, palpitation and insomnia, fatigue, hypodynamia, menorrhea, and anemia	Liang Yao Jia Xiu
6	Dang Shen Dun Rou	Replenish qi and tonifying deficiency, and is suitable for treat asthenia and qi and blood deficiency	Medicinal Diet of China
7	Shen Qi Bu Gao	It is suitable for treating deficiency of both qi and blood as well as women’s menorrhea, fatigue, pale tongue with white coating	Chang Jian Bing De Yin Shi Liao Fa
8	Shen Gui Jiu	Is suitable for qi and blood deficiency, limb fatigue, insomnia, amnesia*etc.*	Xin Bian Zhong Cheng Yao
9	Dang Shen Jiu	Diarrhea due to spleen deficiency, cold limbs, weakness of four limbs, and poor appetite	Proved Recipe
10	Dang Shen Black Tea	Regulating human immunity, and is suitable for health promotion of people with qi and blood deficiency	Lei et al. (2022)
11	Dangshen Beixing Bao Zhufei	Treat chronic bronchitis and cough	Miao Yong Dangshen Zhi Bai Bing
12	Dangshen chrysanthemum Tea	Using the warmth of codonopsis pilosula to alleviate the coldness of chrysanthemum, and has the function of invigorating qi and nourishing blood	Miao Yong Dangshen Zhi Bai Bing

## 4 Phytochemistry

CR contains a variety of chemical components, mainly including alkaloids, alkynes and polyacetylenes, flavonoids, lignans, steroids, terpenoids, organic acids, volatile oils, sugars and other components. In addition, CR is also rich in amino acids, inorganic elements and trace elements required by the human body (Zhan et al., 2021). In this section, we summarize the main chemical constituents of CR in Table 4.

**TABLE 4:** The main chemical constituents of CR.

Type	Compound	Ref
alkynes, polyacetylenes and their glycosides	lobetyol	Mei et al. (2008)
	lobetyolin	Mei et al. (2008)
	lobetyolinin	Ishida et al. (2008)
	Pilosulyne A	Chao et al. (2015)
	Pilosulyne B	Chao et al. (2015)
	Pilosulyne C	Chao et al. (2015)
	Pilosulyne D	Chao et al. (2015)
	Pilosulyne E	Chao et al. (2015)
	Pilosulyne F	Chao et al. (2015)
	Pilosulyne G	Chao et al. (2015)
	Cordifolioidyne A	He et al. (2014)
	Cordifolioidyne B	He et al. (2014)
	Cordifolioidyne C	He et al. (2014)
	Choushenpilosulyne A	Xiao-Yu Hu et al. (2018)
	Choushenpilosulyne B	Xiao-Yu Hu et al. (2018)
	Choushenpilosulyne C	Xiao-Yu Hu et al. (2018)
	Choushenpilosulyne D	Su et al. (2021)
	Choushenpilosulyne E	Su et al. (2021)
	Choushenpilosulyne F	Su et al. (2021)
	Choushenpilosulyne G	Su et al. (2021)
	Codonopilodiynoside A	Jiang et al. (2015a)
	Codonopilodiynoside B	Jiang et al. (2015a)
	Codonopilodiynoside C	Jiang et al. (2015a)
	Codonopilodiynoside D	Jiang et al. (2015a)
	Codonopilodiynoside E	Jiang et al. (2015a)
	Codonopilodiynoside F	Jiang et al. (2015a)
	Codonopilodiynoside G	Jiang et al. (2015a)
	Codonopilodiynoside H	Jiang et al. (2015b)
	Codonopilodiynoside I	Jiang et al. (2015b)
	Codonopilodiynoside J	Jiang et al. (2015b)
	Codonopilodiynoside K	Jiang et al. (2015b)
	Codonopilodiynoside L	Jiang et al. (2015b)
	Codonopilodiynoside M	Jiang et al. (2015b)
	Tangshenyne A	Sun et al. (2016)
	Tangshenyne B	Sun et al. (2016)
	Codonopiloenynenoside A	Jiang et al. (2015b)
	Codonopiloenynenoside B	Jiang et al. (2015b)
	tetradeca-4E, 8E, 12Etriene-10-yne-1, 6, 7-triol	Lin et al. (2013)
	(+)-(6R, 7R, 12E)-tetradeca-12-en-10-yne-1, 6, 7-triol	Lin et al. (2013)
	(2E, 6E)-octa-2, 6-dien-4-ynoic acid	Lin et al. (2013)
	E) -oct-6-en-4-ynoic acid	Lin et al. (2013)
	(+)-(2R, 7S)-1, 7-dihyrdroxy-2, 7-cyclotetradeca-4, 8, 12-trien-10-yn-6-one	Lin et al. (2013)
	9-(tetrahydropyran-2-yl)-non-trans-2, 8-diene-4, 6-diyn-1-ol	Cao (2012)
	9-(tetrahydropyran-2-yl)-non-trans-8-ene-4, 6-diyn-1-ol	Feng et al. (2017)
	pilosulinene A	Xiao-Zhen Li et al. (2019)
	pilosulinol A	Xiao-Zhen Li et al. (2019)
	pilosulinol B	Xiao-Zhen Li et al. (2019)
	pratialin B	Jiang et al. (2015b)
	(-)-(8R, 9R, 2E, 6E, 10E)-tetradeca-2, 6, 10-triene-4-yne-8, 14-diol-9-β-D-glucopyranoside	Wang et al. (2017a), Wang et al. (2017b)
Flavonoids	luteolin	Zhou et al. (2012)
	Luteoloside	Zhou et al. (2012)
	Choushenflavonoids A	Qin et al. (2018b)
	Choushenflavonoids B	Qin et al. (2018b)
	chrysoeriol	A et al. (2012)
	tricin	A et al. (2012)
	apigenin	A et al. (2012)
	apigenin-7-O-β-D-glucopyranoside	Bian et al. (2022)
	luteolin-7-O-β-D gentiobioside	A et al. (2012)
	luteolin-7-O-β-D-glucopyranosyl-(1→6)-(6‴-O-caffeoyl)-β-D-glucopyranoside	He et al. (2014)
	hesperidin	Qi et al. (2011)
	neokurarinol	Xiao-qing Ma et al. (2014)
	kaempferol	Bian et al. (2022)
	wogonin	Bian et al. (2022)
	quercetin	Bian et al. (2022)
	cordifoliflavane A	Qi et al. (2020)
	cordifoliflavane B	Qi et al. (2020)
	5-hydroxy-4', 6, 7-trimethoxy flavone	Fan (2011)
	5-hydroxy-4', 7-dimethoxy flavone	Qi et al. (2020)
	tectoridin	Qi et al. (2020)
	5, 7, 3'5'-tetrahydroxy-flavone-7-O-β-Dglucopyranoside	Qi et al. (2020)
	Choushenoside A	Qin et al. (2018a)
	Choushenoside B	Qin et al. (2018a)
	Choushenoside C	Qin et al. (2018a)
Lignans	tangshenoside Ⅰ	Mizutani et al. (1988)
	tangshenoside ⅠI	Mizutani et al. (1988)
	tangshenoside ⅠII	Mizutani et al. (1988)
	tangshenosideIV	Mizutani et al. (1988)
	tangshenosideV	Mizutani et al. (1988)
	tangshenosideVⅠ	Mizutani et al. (1988)
	tangshenoside VIII	Ren et al. (2013)
	lanceolune A	Hu et al. (2012b)
	lanceolune B	Hu et al. (2012b)
	lanceolune C	Hu et al. (2012b)
	cordifoliketone A	Hu et al. (2012a)
	cordifoliketone B	Hu et al. (2012a)
	coniferoside	Wang et al. (2017a), Wang et al. (2017b)
	syringarsinol	Mao et al. (2000)
	ethylsyringin	A et al. (2012)
	syringing	Wang et al. (2017a), Wang et al. (2017b)
	methlysyringin	Liang et al. (2007)
	lariciresinol	Jiang et al. (2016)
	dehydrodieoniferyl alcohol	Feng et al. (2017)
	balanophonin	Jiang et al. (2016)
	epipinoresinol	Jiang et al. (2016)
	(-)-secoisolariciresinol	Jiang et al. (2016)
	medioresinol	Jiang et al. (2016)
	pinoresinol	Jiang et al. (2016)
	(+)-demethoxypinoresinol	Jiang et al. (2016)
	(-)-(7R,7'R,8R,8'S)-4,4'-dihydroxy-3,3',5,5',7-pentamethoxy-2,7'-cyclolignane	Jiang et al. (2016)
	(-)-ent-isolariciresinol	Jiang et al. (2016)
	(-)-(7R,8S)-dihydrodehydrodiconiferylalcohol-4-O-β-D-glucopyranosyl-(1‴→2'')-β-Dglucopyranoside	Jiang et al. (2016)
	(-)-(7R, 8S)-dihydrodehydrodiconiferyl alcohol	Jiang et al. (2016)
	(+)-(7S, 8S) -3-methoxy-3', 7-expoxy-8,4'-neolignan-4, 9, 9'-triol	Jiang et al. (2016)
	(-)-(7R, 8S, 7'E) -3', 4-dihydroxy-3-methoxy-8,4'-oxyneoligna-7'-ene-7, 9, 9'-triol	Feng et al. (2017)
Steroids	α-spinasterol	Chen et al. (1985)
	β-daucosterol	Qi et al. (2011)
	β-sitosterol	Zhong and Liang (2006)
	Δ5, 25-stigmasterol	KE-ke (2008)
	stigmasterol	Chen et al. (1985)
	α-spinatsrol-β-D-glucoside	Wang and Wang (1996)
	Δ^7^-stigmasterol	Chen et al. (1985)
	Δ^7^ -stigmasteryl-β-D-glucoside	Wang et al. (1986)
	α-spinasterone	Wang and Wang (1996)
	Δ^5,22^-stigmasteryl-β-D-glucoside	KE-ke (2008)
	α-spinasta −7, 22-diene -3-one	KE-ke (2008)
	stigmasta-5,22-diene-3-one	KE-ke (2008)
	Δ5,22-stigmasterol	Gao et al. (2018)
	Δ^5,22^-stigmasteryl-β-D-glucoside	Gao et al. (2018)
	sitosterol	Gao et al. (2018)
Terpenes	taraxeryl acetate	Ishida et al. (2008)
	taraxerol	Gao et al. (2018)
	taraxerone	Mao et al. (2000)
	β-amyrin acetate	Zheng et al. (2018)
	rubiprasin B	Zheng et al. (2018)
	pseudolarolide U	Zheng et al. (2018)
	Pseudolarolide V	Zheng et al. (2018)
	5-hydroxymethyl-2-furaldehyde	Li et al. (2009)
	codonopilate A	Xie et al. (2017)
	codonopilate B	Xie et al. (2017)
	codonopilate C	Xie et al. (2017)
	24-methylenecycloartanyl linolate	Wakana et al. (2011)
	24-methylenecycloartan-3-ol	Wakana et al. (2011)
	friedelin	Wang et al. (1982)
	cycloartenol	Qi et al. (2020)
	oleanolic acid	Liang et al. (2007)
	Codonoposide	Lee et al. (2002)
	Codonolaside	(Zhong and Liang)
	lancemasides A	Ushijima et al. (2008)
	lancemasides B	Ushijima et al. (2008)
	lancemasides C	Ushijima et al. (2008)
	lancemasides D	Ushijima et al. (2008)
	lancemasides E	Ushijima et al. (2008)
	lancemasides F	Ushijima et al. (2008)
	lancemasides G	Ushijima et al. (2008)
	codonolaside Ⅰ	Xu et al. (2008)
	codonolaside ⅠI	Xu et al. (2008)
	codonolaside ⅠII	Xu et al. (2008)
	codonolaside ⅠV	Xu et al. (2008)
	codonolaside V	Xu et al. (2008)
	acid-3-β-D-glucopyranosyl methyl furfural	Xie et al. (2020a), Xie et al. (2020b)
	foetidissimoside A	Ichikawa et al. (2008)
	astersaponin Hb	Ichikawa et al. (2008)
	echinocystic acid-3-O-β-D-glucuronopyranoside	Liang et al. (2007)
	lupeol	Cheng (2010)
	zeorin	Huang et al. (2018)
	codonopsesquiloside A	Jiang et al. (2016)
	codonopsesquiloside B	Jiang et al. (2016)
	codonopsesquiloside C	Jiang et al. (2016)
	friedelan-3-one	Wakana et al. (2011)
	1-friedelan-3-one	Wakana et al. (2011)
	syringaldehyde	Wang et al. (1988)
	Atractylenolide Ⅱ	Wang et al. (1988)
	Atractylenolide ⅡI	Jiang et al. (2016)
	Condonoside A	Tsai and Lin (2008)
	Condonoside B	Tsai and Lin (2008)
Organic acids	caffeic acid	Jing et al. (2013)
	vanillic acid	Wang et al. (1988)
	nicotinic acid	Wang et al. (1988)
	succinic acid	Zhu et al. (2001)
	4-(β-D-glucopyranosyl)-benzoic acid	A et al. (2012)
	shikimic acid	Mao et al. (2000)
	Phenylacetic acid	Su (2018)
	2-furancarboxylic acid	Cao (2012)
	protocatechuic acid	Su (2018)
	fumalic acid	Meng et al. (2003)
	syringic acid	Su (2018)
	codopiloic acid	Wang et al. (1991)
	4-hydroxybenzoic acid	A et al. (2012)
	chlorogenic acid	A et al. (2012)
	neochlorogenic acid	A et al. (2012)
	8-O-4' diferulic acid	Kan (2009)
	maleic acid	Gao et al. (2018)
	linoleic acid	Jing et al. (2013)
	myristic acid	Jiang and Xu (2018)
	stearic acid	Sun et al. (2015)
	lauric acid	Gao et al. (2018)
	9, 12, 13-trihydroxy-10, 15-octadecadienoic acid	An et al. (2018)
	9,12, 13-trihydroxy-10-loctadecarene acid	An et al. (2018)
	9,10-dihydroxy-12-octadecenoic acid	An et al. (2018)
	coronaric acid	Xiao-qing Ma et al. (2014)
	codopiloic acid	Gao et al. (2018)
	(Z)-2-(β-glucopyranosyloxy)-3-phenylpropenoic acid	Tsai and Lin (2008)
	Azelaic acid	Xiao-qing Ma et al. (2014)
	gentisic acid	Su (2018)
	3-O-caffeoylquinic acid methyl ester	Fan et al. (2011)
	3-O-caffeoylquinic acid butyl ester	Fan et al. (2011)
	9,10,13-trihydroxy-(E)-11-octadecenoic acid	Qi et al. (2011)

### 4.1 Alkaloids and nitrogen compounds

Alkaloids are an important class of natural organic compounds, which are cyclic compounds containing negative oxidation state nitrogen ions and present in biological organisms, including pyrroles, piperidines, tropanes, *etc.* The structures are shown in Supplementary Figure S1.

### 4.2 Alkynes, polyacetylenes and their glycosides

Alkynes and polyacetylenes are widely distributed in Campanulaceae plants. What’s more, the Chinese Pharmacopoeia also uses lobetyolin as an index component for the quality evaluation of CR. Studies have shown that in addition to anticancer, antibacterial and anti-inflammatory effects, lobetyolin also have good protective effects against gastric mucosal damage (Gao et al., 2018; Xie and Wang, 2022). The structures of Alkynes, Polyacetylenes and their glycosides are shown in Supplementary Figure S2.

### 4.3 Flavonoids

Flavonoids refer to a series of compounds consisting of two benzene rings with phenolic hydroxyl groups interconnected by three central carbon atoms—compounds composed of C_6_-C_3_-C_6_ units. It is widely distributed in the plant kingdom and has a variety of physiological activities. In addition, it has antioxidant, antiviral, hepatoprotective, anticancer, anti-inflammatory and other pharmacological activities, and is an important class of natural organic compounds. The flavonoid components isolated from CR are mainly flavones, flavonols and their glycosides. The structures of Flavonoids compounds are shown in Supplementary Figure S3. At present, there are fewer studies on the flavonoid component of CR, and in future studies, we should pay more attention to this component with multiple pharmacological effects.

### 4.4 Lignans and their glycosides

Lignans are defined as a class of natural products with a phenyl propane backbone formed by two structures linked by β, β´ or 8, 8´-carbons therein, usually referring to their dimers and, to a lesser extent, trimers and tetramers.

Tangshenoside I-IV are exclusive components of CR, of which tangshenoside I is about ten times more than the other three glycosides (Mizutani et al., 1988). However, there are still few reports on the pharmacological effects of Tangshenoside I. The structures of lignans are summarized in Supplementary Figure S4. In the following research, we should pay more attention to this exclusive ingredient of CR, and expand the scope of its medical use.

### 4.5 Steroids and their glycosides

Steroids are a naturally widespread class of chemical components, of which there are many types, but all have cyclopentano-perhydrophenanthrene in their structure. The steroids components of CR are sterols, steroidal glycosides and steroids, and the structures of each component are shown in Supplementary Figure S5.

### 4.6 Terpenes and their glycosides

Terpenes are the most abundant class of natural products with a wide distribution, complex skeleton and a variety of biological activities. In addition, terpenes are compounds that have an isoprene unit (C_5_) as the basic structural unit. The terpene components isolated from CR mainly include tetra- and pentacyclic triterpenes, hemi- and sesquiterpenes, and they mostly exist in the form of glycosides. The sesquiterpene lactone compounds atractylenolide II and atractylenolide III were isolated for the first time from CR by Wang ZT et al. and reported for the first time in Campanulaceae (Wang et al., 1988). Atractylenolide III is one of the main components of CR, which has relatively obvious anti-inflammatory effect. Some researchers believe that atractylenolide III can also be used as an index component in the quality evaluation of CR. In this section, we systematically summarize the chemical composition of CR and its structures are summarized in Supplementary Figure S6.

### 4.7 Organic acids

Organic acids are compounds containing carboxyl groups (excluding amino acids) that are widely found in living things. The most common organic acids are carboxylic acids, whose acidity originates from the carboxyl group (-COOH). In addition, sulfonic acid (R-SO_3_H), sulfinic acid (RSOOH) and thiocarboxylic acid (RCOSH) are also organic acids. The organic acid composition in CR is summarized in Supplementary Figure S7.

### 4.8 Volatile oil

Volatile oil, also known as essential oil, is a generic term for a class of oily droplets with an aromatic odor, which contains a complex composition, usually consisting of tens to hundreds of components. Liao J et al. identified 32 components from the volatile oil of CR, 11 acidic components, mainly brown shackle acid, and 21 neutral components (Liao and Lu, 1987); Li Cong et al. used GC-MS coupling technique to isolate 66 components from the volatile components of CR, and identified 50 chemical components by Wiley and NBS standard spectral library search (Li et al., 1993); Guo QQ used steam distillation and headspace gas chromatography to extract volatile components of CR for GC-MS analysis, and 117 volatile compounds were detected (Guo, 2016). The volatile oil components of CR are mainly alcohols, aldehydes, terpenes, ketones, furans, acids, olefins, alkanes, phenols, sulfides and other compounds. In this paper, we compiled 120 volatile oil components extracted from CR, as shown in Table 5.

**TABLE 5:** The volatile oil components of CR.

NO.	Type	Compounds	Molecular formula	Ref
1	alcohols	1,2,3,4-Butanetetrol	C_10_H_10_O_4_	Guo (2016)
2		1-Pentanol	C_5_H_12_O	Guo (2016)
3		3-Hexen-1-ol	C_6_H_12_O	Guo (2016)
4		2-Hexen-1-ol	C_6_H_12_O	Guo (2016)
5		1-Hexanol	C_6_H_12_O	Guo (2016)
6		4-Hexen-1-ol	C_6_H_12_O	Guo (2016)
7		1-Octen-3-ol	C_8_H_16_O	Guo (2016)
8		Benzyl Alcohol	C_7_H_8_O	Guo (2016)
9		1-Octanol	C_8_H_18_O	Guo (2016)
10		3,7-dimethyl-1,6-Octadien-3-ol	C_10_H_18_O	Guo (2016)
11		Z-2-Tridecen-1-ol	C_13_H_26_O	Guo (2016)
12		borneol	C_10_H_18_O	Liao and Lu (1987)
13		(+)-γ-costol	C_15_H_24_O	Li et al. (1993)
14		Hexanol	C_4_H_14_O	Li et al. (1993)
15	aldehydes	2-methyl-Propanal	C_4_H_8_O	Guo (2016)
16		3-methyl- Butanal	C_5_H_10_O	Guo (2016)
17		2-methyl- Butanal	C_5_H_10_O	Guo (2016)
18		Pentanal	C_5_H_10_O	Guo (2016)
19		2-Ethylacrolein	C_5_H_8_O	Guo (2016)
20		Hexanal	C_6_H_12_O	Guo (2016)
21		(Z)-3-Hexenal	C_6_H_10_O	Guo (2016)
22		2-Hexenal	C_6_H_10_O	Guo (2016)
23		5-methyl-Hexanal	C_7_H_14_O	Guo (2016)
24		Heptanal	C_7_H_14_O	Guo (2016)
25		(E, E)-2,4-Hexadienal	C_6_H_8_O	Guo (2016)
26		Benzaldehyde	C_7_H_6_O	Guo (2016)
27		Octanal	C_8_H_16_O	Guo (2016)
28		(E, E)-2,4-Heptadienal	C_7_H_10_O	Guo (2016)
29		Benzeneacetaldehyde	C_8_H_8_O	Guo (2016)
30		Nonanal	C_9_H_18_O	Guo (2016)
31		(E, Z)-2,6-Nonadienal	C_9_H_14_O	Guo (2016)
32		((E)-2-Nonenal	C_9_H_16_O	Guo (2016)
33		2,5-Thiophenedicarboxaldehyde	C_6_H_4_O_2_S	Guo (2016)
34		4-methoxy-Benzaldehyde	C_8_H_8_O_2_	Guo (2016)
35		(E, E)-2,4-Decadienal	C_10_H_16_O	Guo (2016)
36		2-butyl-2-Octenal	C_12_H_22_O	Guo (2016)
37		2-Isopropylidene-3-methylhexa-3,5-dienal	C_10_H_14_O	Guo (2016)
38		4-hydroxy-3,5-dimethoxy-Benzaldehyde	C_9_H_10_O_4_	Guo (2016)
39		(Z)-9,17-Octadecadienal	C_18_H_32_O	Guo (2016)
40		2-furancarboxaldehyde	C_5_H_4_O_2_	Li et al. (1993)
41		p-Methoxy benzaldehyde	C_8_H_8_O_2_	Li et al. (1993)
42	terpenes	Limonene	C_10_H_16_	Guo (2016)
43		α-gurjunene	C_15_H_24_	Guo (2016)
44		2-Methylbicyclo [4.3.0] non-1 (6)-ene	C_10_H_16_	Guo (2016)
45		β-cedrene	C_15_H_24_	Guo (2016)
46		Caryophyllene	C_15_H_24_	Guo (2016)
47		Thujopsene	C_15_H_24_	Guo (2016)
48		γ-Elemene	C_15_H_24_	Guo (2016)
49		(E)-β-farnesone	C_15_H_24_	Li et al. (1993)
50		β-selinene	C_15_H_24_	Li et al. (1993)
51		(-)-β-Acoradiene	C_15_H_24_	Li et al. (1993)
52		γ-Muurodone	C_15_H_24_	Li et al. (1993)
53		2,6-dimethyl-2,4,6-Octatriene	C_15_H_24_	Guo (2016)
54		Cedrene	C_15_H_24_	Guo (2016)
55		chamigrene	C_15_H_24_	Guo (2016)
56		1-methyl-4-(5-methyl-1-methylene-4-hexenyl)-Cyclohexene	C_15_H_24_	Guo (2016)
57		α-Pinene	C_10_H_16_	Liao and Lu (1987)
58		δ-Guaiene	C_15_H_24_	Liao and Lu (1987)
59		β-bisabolene	C_15_H_24_	Li et al. (1993)
60		Iso Longiforenexide	C_15_H_24_O	Li et al. (1993)
61	ketones	Benzocyclobuten-1(2H)-one	C_8_H_6_O	Guo (2016)
62		Benzocyclobuten-1(2H)-one	C_8_H_6_O	Guo (2016)
63		2,3-Octanedione	C_8_H_14_O_2_	Guo (2016)
64		6-methyl-5-Hepten-2-one	C_8_H_14_O	Guo (2016)
65		1-(4-methylphenyl)-Ethanone	C_9_H_10_O	Guo (2016)
66		Atractylon	C_15_H_20_O	Li et al. (1993)
67		Methyl caproate	C_7_H_14_O_2_	Guo (2016)
68		Hexyl caproate	C_12_H_24_O_2_	Guo (2016)
69		Ethyl palmitate	C_18_H_36_O_2_	Guo (2016)
70		Linoleic acid ethyl ester	C_20_H_36_O_2_	Guo (2016)
71		methyl caprylate	C_9_H_18_O_2_	Xie et al. (2000)
72		methyl stearate	C_19_H_38_O_2_	Liao and Lu (1987)
73		Methyl tetradecanoate	C_15_H_30_O_2_	Liao and Lu (1987)
74		Methyl Pentadecanoate	C_16_H_32_O_2_	Liao and Lu (1987)
75		Methyl Hexadecanoate	C_17_H_34_O_2_	Liao and Lu (1987)
76		Methyl octadecarbodienoate	C_19_H_34_O_2_	Liao and Lu (1987)
77		diene steariate	C_20_H_36_O_2_	Xie et al. (2000)
78		1,2-Benzenedicarboxylic acid, mono (2-ethylhexyl) ester	C_16_H_22_O_4_	Guo (2016)
79	furans	2-ethyl-Furan	C_6_H_8_O	Guo (2016)
80		2-pentyl-Furan	C_9_H_14_O	Guo (2016)
81		trans-2-(2-Pentenyl) furan	C_9_H_12_O	Guo (2016)
82	acids	Acetic acid	C_2_H_4_O_2_	Guo (2016)
83		Hexanoic acid	C_6_H_12_O_2_	Guo (2016)
84		Non-anoic acid	C_9_H_18_O_2_	Guo (2016)
85		heptanoic acid	C_7_H_14_O_2_	Xie et al. (2000)
86		tetradecanoic acid	C_14_H_28_O_2_	Xie et al. (2000)
87		pentadecanoic acid	C_15_H_30_O_2_	Xie et al. (2000)
88		Hexadecylic acid	C_16_H_32_O_2_	Xie et al. (2000)
89		Heptede canoic acid	C_17_H_34_O_2_	Li et al. (1993)
90		non-adecane	C_19_H_40_	Xie et al. (2000)
91		(Z, Z)-9,12-Octadecadienoic acid	C_18_H_32_O_2_	Guo (2016)
92		Adamantane acid	C_12_H_18_O_2_	Li et al. (1993)
93	olefins	Cyclohexene	C_6_H_10_	Guo (2016)
94		1-(1,5-dimethyl-4-hexenyl)-4-methyl- Benzene	C_15_H_22_	Guo (2016)
95		Squalene	C_30_H_50_	Guo (2016)
96	alkanes	Tridecane	C_13_H_28_	Guo (2016)
97		Tetradecane	C_14_H_30_	Guo (2016)
98		pentadecane	C_15_H_32_	Li et al. (1993)
99		Hexadecane	C_16_H_34_	Guo (2016)
100		Heptadecane	C_17_H_36_	Liao and Lu (1987)
101		octadecane	C_18_H_38_	Liao and Lu (1987)
102		9-methyl-Nonadecane	C_20_H_42_	Guo (2016)
103		Eicosane	C_20_H_42_	Liao and Lu (1987)
104		n-heneicosane	C_21_H_44_	Liao and Lu (1987)
105		n-docosane	C_22_H_46_	Liao and Lu (1987)
106	phenols	3-methyl phenol	C_7_H_8_O	Li et al. (1993)
107		2-Methoxy phenol	C_7_H_8_O_2_	Li et al. (1993)
108		4 Ethyl Phenol	C_8_H_10_O	Li et al. (1993)
109		3,5-Dinethyl Phenol	C_8_H_10_O	Li et al. (1993)
110		2,6-Dimethoxy-4 (2-Propenyl)-Phenol	C_11_H_14_O_3_	Li et al. (1993)
111	sulfides	Dimethyl sulfide	C_2_H_6_S	Guo (2016)
112		dimethyl Disulfide	C_2_H_6_S_2_	Guo (2016)
113	Others	2-methoxy-3-(1-methylethyl)- Pyrazine	C_8_H_12_N_2_O	Guo (2016)
114		Naphthalene	C_10_H_8_	Guo (2016)
115		Benzothiazole	C_7_H_5_NS	Guo (2016)
116		1-methyl-Naphthalene	C_11_H_10_	Guo (2016)
117		pentachloro-Benzene	C_6_HCl_5_	Guo (2016)
118		Caryophyllene oxide	C_15_H_24_O	Guo (2016)
119		Phenanthrone	C_14_H_10_	Li et al. (1993)
120		Anthracene	C_14_H_10_	Li et al. (1993)

### 4.9 Saccharides

Saccharides are widely found in plants, and ginseng is also rich in saccharides, including monosaccharides, oligosaccharides and polysaccharides, *etc.* Moreover, Codonopsis polysaccharides (CPPs) is one of the main components of CR, mainly composed of five-carbon sugar, six-carbon sugar and its derivatives, which has good central inhibitory effect and anti-ulcer effect (Zhao, 2016). We have systematically summarized the saccharides compounds of CR, the methods of extraction, separation and purification, monosaccharide composition and analysis method, as shown in Table 6.

**TABLE 6:** The saccharides compounds of CR, the methods of extraction, separation and purification, monosaccharide composition and analysis method.

Compounds	Extraction, separation and purification methods	Monosaccharide composition	Analysis method	Ref
CPPS1	Extraction: water extraction and alcohol precipitation; Separation and purification: Sevage method for deproteinization, DEAE-cellulose column chromatography	arabinose, ribose, mannose, fructose, galactose, glucose	GC	Ren et al. (2008)
WCP-Ia	Extraction: water extraction and alcohol precipitation Separation and purification: concentration, dialysis to remove NaCl, lyophilization	Galacturonic acid, galactose, rhamnose, arabinose	GC	Zou et al. (2019)
Homogeneous polysaccharide RCNP, RCAP-1 and RCAP-2	Extraction: water extraction and alcohol precipitation Separation and purification: DEAE 650 M, Superdex G-200	RCNP contains arabinose and arabinogalactan; RCAP-1 and RCAP2 are highly methyl esterified pectin-type polysaccharides	HPGPC, Chemical derivative analysis method, GC–MS and NMR	Sun et al. (2019)
CPA, CP-B, CPC	Extraction: water extraction and alcohol precipitation; Separation and purification: column chromatography, gel filtration, ultracentrifugation and membrane separation techniques	CPC is a dextran	HPGPC, UV, IR, Acid hydrolysis, methylation analysis, GC-MS, NMR	Wang (2021)
CPP-2-1, CPP-2-2, CPP-3-1, CPP-3-2	Extraction: water extraction and alcohol precipitation Separation and purification: ion-exchange chromatography and Sepharose CL-6B	Rhamnose, galacturonic acid, arabinose, galactose, glucose, mannose	HPLC	Zhu (2013)
CPP1-2-1	Extraction: water extraction and alcohol precipitation Separation and purification: Sephadex G-150, Sephadex G-100	inulin-type fructan	TLC, GC-MS.	Meng et al. (2020)

### 4.10 Others

In addition to the above chemical constituents, other chemical compositions of CR are listed in Table 7, and the structures are shown in Supplementary Figure S8. In addition, CR contains many amino acids, such as glutamic acid, glycine, tyrosine, serine and other 15 amino acids, as well as Ca, Mg, Zn, Fe, Cu, Mn and other 33 inorganic elements. Among them, the content of nutrients and beneficial elements (such as P, Ca, Mg, Fe, Zn, *etc.*) required by human body is high, while the content of harmful elements (such as Pb, Cd, AS, Hg, *etc.*) is low (Wang et al., 2011; Sun et al., 2015).

**TABLE 7:** Other components of CR.

NO.	Compounds	Ref
1	angelicin	Zhu et al. (2001)
2	psoralen	Zhu et al. (2001)
3	5-hydroxymethyl-2-furaldehyde	Wang et al. (1988)
4	2-furaldehyde	He et al. (2006)
5	emodin	He et al. (2006)
6	geniposide	He et al. (2006)
7	sweroside	Cheng (2010)
8	eucommioside II	Xie et al. (2020a), Xie et al. (2020b)
9	(6R,9S)-3-oxo-α-ionol-β-D-glucopyranoside	Xie et al. (2020a), Xie et al. (2020b)
10	woodorien	Xie et al. (2020a), Xie et al. (2020b)
11	1,3-linolein-2-olein	Fan et al. (2011)
12	genipin gentiobioside	Xie et al. (2020a), Xie et al. (2020b)

In this section, we provide a detailed summary of the chemical constituents obtained from the current extraction and isolation of CR. Currently, more than 400 compounds are obtained from CR, including 120 volatile oil components. In addition, the traditional medicinal part of CR is the root, and the above-ground part is less studied, while the above-ground part contains a variety of chemical components with high utilization value, but its utilization and research are only in the primary stage, and the reasonable and effective exploitation has not yet been carried out, resulting in a serious waste of resources. In the next research, we should study other parts of CR, such as the leaves and stems of it, in order to enhance the utilization of non-traditional medicinal parts of medicinal plants and carry out a deeper comprehensive utilization of resources to avoid wasting resources.

## 5 Analysis method and quality control

### 5.1 Analysis method

There are many chemical components and various methods of determination of CR. With the development of modern technology, the instruments are also becoming more sensitive, precise and accurate. In this paper, we have compiled the analytical methods of CR in recent years in order to help scholars to better develop and utilize CR and establish quality control standards for CR. Phenol-sulfuric acid method is the main method for determination of polysaccharide content, scholars used UV-300 ultraviolet spectrophotometer, 721 spectrophotometer and phenol-sulfuric acid colorimetric method to analyze the polysaccharides of CR (Wang et al., 1993). Analysis of total saponins of Codonopsis (TSC) and CPPs of CR by ultraviolet spectrophotometry (UV-Vis) by Dou et al. (2015); Dou et al. (2016). Zhen X et al. used UV spectrophotometric method to determine CPPs of Lu Dangshen and other origins of CR and found that the average recovery was 97.56%, and the average value of CPPs of CR was 18.39%, which provided the basis for the quality standard of Lu Dangshen (Zhen et al., 2014). The method is characterized by higher sensitivity, better accuracy, simple instrumentation, easy operation and faster analysis, but lacks selectivity for related substances with similar structures. The volatile oil components are often determined by gas chromatography, Guo analyzed the volatile components of CR by Head space gas chromatography-mass spectrometry (HS-GC-MS) to search for the main volatile components with special aroma in CR (Guo, 2016). The pretreatment of this method is simple and the cost of the instrument is low, but it requires the substance to be measured to have volatility. Therefore, this method has certain limitations and narrow application scope.

At present, the chemical constituents of CR are mostly studied by HPLC, which provides reference for establishing the quality standard of CR. For example, some scholars use HPLC method to analyze the content of lobetyolin in different origins (Song et al., 2008; Shi et al., 2011; Chen H. et al., 2014); Other scholars used HPLC to simultaneously determine the contents of lobetyolin and syringing in CR from different origins (Chen et al., 2016). This method is featured with wide applicability, high separation efficiency, high automation and easy sample recovery. The mobile phase, however, is mostly toxic organic reagent, which will pollute the environment. There are also scholars who evaluate the quality evaluation of Codonopsis Radix and processed products based on the analysis of monosaccharides and oligosaccharides by liquid chromatography coupled with charged aerosol detector, the method is independent of the optical or structural characteristics of the sample and is responsive to samples that do not contain chromophoric groups (Xie et al., 2022). With the enhancement of people’s awareness of environmental protection, supercritical fluid chromatography (UPC^2^) has come into the view of the majority of scholars. It uses supercritical fluid as the mobile phase (supercritical CO_2_ is often used), which has the advantages of both gas chromatography and liquid chromatography, and can achieve rapid and effective separation. Using the UPC^2^ method, Yang Liu et al. screened and quantified the quality markers of *Angelica pubescens Maxim. f. biserrata Shan et Yuan* [Apiaceae, Angelicae Pubescentis Radix], which is greener and safer compared to the traditional chromatographic method (Yang et al., 2020). This can be used as a reference for relevant analytical work in future research on CR. In addition, the higher sensitivity and better accuracy of chromatography-mass spectrometry tandem technique can also effectively reduce the experimental cost. For example, some scholars have established UPLC-MS/MS method to determine the chemical composition of CR (Ma F.et al., 2014; An et al., 2018; Zheng et al., 2021). Compared with the traditional liquid phase method, this method has the advantages of high sensitivity, short analysis time, good selectivity, low detection line and wide application range. However, the instrumentation of the above methods is high-end and expensive. We believe that with the development of science and technology, more low-cost and efficient instruments as well as green reagents will be available, and in the near future, we can use more efficient, accurate and green methods for the research related to CR.

### 5.2 Quality control

CR, a traditional Chinese medicine, has complex chemical components and wide drug sources. As a natural product of medicine and food, people’s demand for CR is increasing day by day. However, there are many kinds of CR, and it is difficult to distinguish them. Moreover, fake and inferior products of CR are often found in the Chinese herbal medicine market. In addition, the quality of Chinese medicinal materials is affected by geographical location, climatic environment, cultivation techniques and other aspects. There are some differences in the quality of medicinal materials from different places of origin, and more importantly, the quality of medicinal materials from the same place of origin is unstable. Due to the above factors, currently, the quality and clinical efficacy of CR cannot be effectively controlled. Therefore, it is necessary for us to use modern science and technology to establish quality control standards for the quality of CR and to evaluate its quality. In this paper, we have compiled literature on quality control of CR from a wide range of scholars in recent years, hoping that it can be helpful for the establishment of quality control standards for CR.

Currently, the 2020 edition of Chinese Pharmacopoeia uses thin-layer chromatography to identify CR, and requires that the moisture content should not exceed 16.0%, the total ash content should not exceed 5.0%, and the sulfur dioxide residue should not exceed 400 mg/kg. Using the hot leaching method to determine, with 45% ethanol as the solvent, the leachate should not be less than 55.0%.

However, there is no content determination item in Chinese Pharmacopoeia, but only characterization and identification items. Chinese medicines are featured with multi-component and multi-target, *etc.* To establish a perfect quality standard research, it is also necessary to clarify the active ingredients or indicator components for Chinese medicines to exert their medicinal effects. Ichikawa et al. simultaneously determined the contents of seven saponins in CR by LC-MS (Ichikawa et al., 2009); Chen J et al. measured the total ash and acid-insoluble ash and determined the content of Atractylenolide III in CR by HPLC (Chen et al., 2012); Kim et al. used HPLC-UV to analyze tangshenoside Ⅰ, lobetyolin and lobetyol to establish the fingerprint profile of CR (Kim et al., 2014); Peng R et al. used HPLC to measure the content of lobetyolin, colorimetric method to determine CPPs and TSC, atomic absorption spectrometry to analyze the contents of As, Pb, Hg, Cd and Cu in CR, gas chromatography to determine BHC and DDT, and the rest of the items were determined by referring to the method included in the Chinese Pharmacopoeia. The results showed that the content of lobetyolin was not less than 0.53 mg g^−1^, CPPs was not less than 14.7%, and the TSC was not less than 7.3 mg g^−1^ (Peng et al., 2014).

In addition, the chemical components of traditional Chinese medicine are complex and diverse, and the therapeutic effect is through the whole of multiple components and multiple targets. The components of CR are diverse and have different pharmacological effects. CPPs has the function of enhancing immunity, Atractylenolide III has the effect of anti-inflammation, and lobetyolin has anti-gastric ulcer pharmacological activity. Therefore, there are certain limitations in applying a single component as a quality control standard to effectively keep the quality of CR. The complex and diverse chemical composition and the unclear pharmacological pathways are the key points and difficulties in the quality evaluation of Chinese medicine. More methods should be built on activity-related or quality evaluation based on chemical activity, and the quality standard and evaluation system that can reflect the core efficacy of CR should be worked out to ensure the scientific, rational and safe use of CR (Qi et al., 2022).

## 6 Processing method

Processing is a major feature of TCM medication and an important part in improving clinical efficacy. According to the needs of the disease, the original properties of the drug are traded off through processing, so that the desired effect is highlighted and other effects are diminished to better suit clinical treatment.

### 6.1 Ancient processing research

The CR has been involved in the mixing of Radix Ginseng for thousands of years. The name of Dangshen and its medicinal method were first recorded in the Qing Dynasty, with a short medicinal history. Further, when used as a medicine, raw Codonopsis Radix is commonly used, and has the effects of replenishing qi and promoting fluid production, which is mostly used for the treatment of spleen and lung qi deficiency, food deficiency and fatigue, as well as qi and blood deficiency. Therefore, its ancient processing method is relatively simple and is occasionally recorded in ancient books.

In terms of purification, the main method used is to “remove the tip” (Qing “Zhi Jin”); “Bamboo knife scraping, violently dried” (Qing dynasty, “Ben Cao Hai Li”); Processing, have “Honey broiling” (Qing dynasty, “Zhi Jin”); Tonifying the lungs, steaming with honey. (Qing Dynasty, “De Pei Ben Cao”); Stir-baking with rice of CR, can cure diarrhea due to deficiency-cold of spleen (Qing Dynasty, “Shi Bing Lun”) (Li et al., 1993; Chen and Fang, 2013). In addition to that above processing methods, there is also the characteristic processing method of CR of Menghe Medical School, including Ginger Juice fried Codonopsis Radix, (Ma Peizhi’s medical case, 马培之医案; He jiheng’s medical case, 贺季衡医案), Agastachis Herba fried Codonopsis Radix (Ma Peizhi’s medical case), Aucklandia Radix fried Codonopsis Radix (Deng Xingbo’s Clinical Medical Collection, 邓星伯临证医集) and so on (Lv et al., 2018; Shen et al., 2023).

In conclusion, the ancient methods of purification of CR include removing the tip and bolting skin, and the methods of processing include honey-roasted CR, steaming with honey, and fried CR with rice.

### 6.2 Modern processing research

The modern common processing methods of CR include fried CR with rice (Wang et al., 2020), honey-roasted CR (Tang et al., 2022), soil fried CR, fried CR with bran and CR processed with wine (Liu et al., 2006). The chemical composition changes and pharmacological effects before and after processing by different processing methods are summarized in Table 8.

**TABLE 8:** The chemical composition changes before and after processing and pharmacological effects by different processing methods.

Processing methods	The chemical composition changes before and after processing	Pharmacological effects	Ref
Fried CR with rice	After fired with rice, the content of that alcohol-soluble extract of CR is obviously reduce; Volatile oil content decreased; Variation in the content of lobetyolin is uncertain	The rice processed CR helps enhance the efficacy of replenishing qi and invigorating spleen	Wang et al. (2020), Tie et al. (2021)
Honey-roasted CR	The total polysaccharide content of CR increase; The content of codonopsis acetylide, atractylodes lactone III and the extract of polysaccharide were all reduced	After honey processing, the function of moistening lung, relieving cough, tonifying the middle and replenishing qi is enhanced	Tang et al. (2022)
Soil fried CR	The content of CPPs decreased; The content of trace elements increased	Enhancing the effects of invigorating spleen and relieving diarrhea	Wang et al. (2018)
bran-processed	The content of CPPs and volatile components decreased	Enhancing effects of harmonizing stomach and invigorating spleen	Wang et al. (2018)
CR processed with wine	The content of lobetyolin and CPPs increased	No studies have been reported	Chen et al. (2019)

Fried CR with rice is the most commonly used method of processing CR, which is included in China Pharmacopoeia (2020 edition). After fried with rice, the smell is burnt and fragrant, which can enhance the effect of invigorating qi and invigorating spleen. At present, the changes of lobetyolin content after rice frying are inconsistent, which may be due to differences in processing technology. Studies have shown that with the increase of frying time, the content of lobetyolin increases firstly and then decreases (Wang M. et al., 2021). So far, the processing technology of CR has mostly continued the ancient method without specific process parameters, and still relies on empirical judgment and does not quantify indicators such as firepower and temperature, which is not conducive to the large-scale processing and production of modern enterprises. Therefore, in the next study, the processing standard of CR should be established, and the factors affecting the quality of CR such as its processing materials, processing time and fire power should be detailed and digitized, and the quality evaluation standard of CR should be established to facilitate the rational clinical application of CR. In addition, the current research on the changes of chemical composition of CR before and after concoction is only limited to CPPs, lobetyolin and volatile oil. Chinese medicines are characterized by multi-target and multi-component combined effects, so the study of only a few components is limited and unspecific. The study of the changes in chemical composition before and after concoction should be more in-depth and comprehensive.

## 7 Pharmacological effects

Modern pharmacological studies have shown that CR has the ability to protect nerve cells (Luo et al., 2017), protect gastrointestinal mucosa and anti-ulcer (Cui et al., 1988), regulate body immunity (Zhang et al., 2003), anti-tumor (Wu et al., 2016), improve hematopoietic function and protect cardiovascular (Li Q. et al., 2019), antibacterial and antiviral (Bai et al., 2015), anti-aging and antioxidant effects (Xu et al., 2006), which have been confirmed by many scholars. These pharmacological effects are summarized in Table 9 and are discussed in detail below.

**TABLE 9:** The pharmacological activity of CR.

Effects	Extract/Compound Dose	Animal/cell line	Study design	Control	Mechanism/results	Ref
Neuroprotection	CPPs, 25,50,100 μmol/L	Sodium thiosulfate-injured neural stem cells	vitro	Blank	CPPs showed significant protective effect on sodium thiosulfate-injured neural stem cells	Wu et al. (2008)
	CPPs,100, 300 mg/kg	hTau-induced behavioral deficits in C57/BL6 mice	vivo	None	CPPs attenuate tau protein hyperphosphorylation by upregulating PP2A activity and rescue hTau-induced behavioral deficits by restoring synaptic plasticity	Zhang et al. (2018)
Neuroprotection	BCP decoction, 0.54, 1.08, 2.16 g kg^-1^·d^-1^	Cognitive dysfunction induced by high activity GSK-3β in rats with Alzheimer’s disease (AD) model	vivo	Water	BCP could effectively improve the cognitive dysfunction induced by elevated GSK-3β activity in rats, and the possible mechanism was related to the downregulation of GSK-3β activity, which in turn inhibited Tau protein hyperphosphorylation and promoted neuronal development	Luo et al. (2017)
	CPPs, 100, 300 mg/kg/d	APP/PS1 mice; HEK293-T cells and mouse neuroblastoma N2a cells	Vitro and vivo	Saline	One-month intragastric administration of CPPs attenuated cognitive impairments in APP/PS1 mice	Wan et al. (2020)
	CPPs, 50 μg/mL	Aβ_1-40_-induced PC_12_ cells model	vitro	blank	CPPs ameliorated Aβ_1-40_-induced neuronal injury, and that its mechanisms may be *via* downregulation of CD38 expression, recovery of NAD + levels, and promotion of mitochondrial ATP synthesis	Hu et al. (2021)
	CPPs, 150, 300 mg kg^-1^	Impaired memory consolidation in mice caused by intraperitoneal injection of cycloheximide	vivo	piracetam	CPPS can improve the memory consolidate obstacle induced by cyclohexanol imide, which related to raise the protein expression of CaMKⅡ/CREB signaling pathway	Mei et al. (2020)
Protect gastrointestinal mucosa and anti-ulcer	CPPS, 250, 500 mg/kg	Four rat gastric ulcer models (stress type, anti-inflammatory pain type, acetic acid type, pylorus ligation type)	vivo	Distilled water	It had a significant inhibitory effect on the increase of gastric acid caused by pilocarpine, while the content of PGE2 in gastric juice increased	Cui et al. (1988)
	the decoction of codonapsis pilosula, 0.8 g/kg	normal	vivo	Distilled water	CR increases the content of growth inhibitors in the gastric and duodenal mucosa of rabbits	Wei Chen et al. (2002)
	The water exart of CR, 5.10 g kg^-1^·d^-1^	Water immersion restraint stress ulcer rat model	vivo	epidermal growth factor	The water extract of CR can alleviate gastric mucosa damage of stress ulcer rat, alleviate pathological damage, and increase polyamine (spermidine) content of gastric mucosa	Li et al. (2013)
	Codonopsis Radix flavonoids	intestinal endothelial cells 6	vitro	blank	Promotion of cell migration is associated with effects on polyamine-regulated signaling pathways	Li R. et al. (2014)
	CPPs, 50,100,200 mg kg^-1^	normal	vivo	The concentrate of Sijunzi decoction	Promotes small intestinal propulsion in mice, growth and digestive capacity in rats	(Xiao-qing Ma et al., 2014)
	BCP, 0.25,0.50,1.00 g/kg	Trinitrobenzene sulfonic acid (TNBS)/ethanol enema induced ulcerative colitis disease model in rats; lipopolysaccharide (LPS) induced inflammation model in mouse mononuclear macrophages (RAW264.7)	vivo and vitro	Guchang Zhixie Pills	The mechanism of therapeutic effect of BCP on UC may be through the activation of TLR4-NF-κB pathway to regulate the secretion of cellular inflammatory factors (IL-6, TNF-α and IL-10) and inflammatory mediators (NO)	Zhou et al. (2016)
Protect gastrointestinal mucosa and anti-ulcer	CP-A, 25,50 mg/kg	ethanol-induced acute gastric ulcer in rats	vivo	bismuth potassium citrate	CP-A were likely the potential component in Radix Codonopsis for treatment of acute gastric ulcers.	Jiankuan Li et al. (2017)
	The water extract of CR, 5,10,15 g/kg	The aging model of mice was induced by D-gal	vivo	Saline	CR can interfere with aging in a holistic manner and protect the gastrointestinal tract of aging mice	Meng (2021)
	TSC, 0.4,1.2 g/kg	Combined TNBS/ethanol enema for rat UC model	vivo	Salazosulfadiazin (SASP)	TSC had a significant protective effect on colonic mucosal injury in UC rats, which was better in the high dose group; the mechanism may be related to anti-lipid peroxidation, inhibition of NF-κB signaling pathway thus regulating the release of inflammatory factors	Liu et al. (2021)
Adjust the immune function and enhance the immunity of the body	Ludangshen oral liquid, 3.3,6.6,13.2 g·kg-1	Establishment of immunosuppressed mouse model by intraperitoneal injection of cyclophosphamide	vivo	Lentinan	CPP inhibits breast cancer cell proliferation and induces apoptosis through downregulation of CCHE1	Tianhui et al. (2019)
	CPPs, 50,100,200 mg/kg	Cyclophosphamide (CTX) induced immune deficiency in a rat model	vivo	Saline	The immunomodulatory activity of CPP in immunocompromised rats may be related to the inhibition of the expression of apoptosis-related proteins Bax and NF-κBp-p65 in immune organs and the upregulation of the expression of anti-apoptosis-related protein Bcl-2	Xu (2018)
	C. pilosula oligosaccharides	Cyclophosphamide induced immunosuppression in mice	vivo	thymosin	CPO might exert immunomodulatory effects through the MAPK signaling pathway	Bai et al. (2020)
	RCAP-1, RCAP-2, 1.6,8,40,200,1000 μg/mL	The murine macrophage cell line RAW 264.7	vitro	blank	RCAP-1 and RCAP-2 exerted a significant immunostimulatory effect based on NO production from RAW264.7 macrophages	Sun et al. (2019)
Anti-tumor	Codonoposide 1c, 20,40 μM	HL-60 human promyelocytic leukemia cells	vitro	β-Actin	Codonoposide 1c induced apoptotic cell death in HL-60 cells	Lee et al. (2005)
	Lancemaside D, 10–100 μg/mL	Human liver cancer cell line HepG-2	vitro	Blank	Inhibition of proliferation of human hepatocellular carcinoma HepG-2 cells	Li (2012)
	TSC, 100–2000 mg/L	human hepatocellular carcinoma SMMC-7721 cells	vitro	Blank	Inhibitory effect on human hepatocellular carcinoma SMMC-7721 cells	Fang et al. (2015)
	CPPs,50、100、200、400 μg/mL	HepG2 cells	vitro	Blank	CPP1a and CPP1c could induce apoptosis through up-regulating the ratio of Bax/Bcl-2 and activating caspase-3	Bai et al. (2018)
	CPP1b,50–400 μg/mL	A549 cells	vitro	inositol	CPP1b exhibited obvious cytotoxicity to human lung adenocarcinoma A549 cells	Chen et al. (2014b)
	the water extract of CR	GPL model	vivo	Saline	CR exerted anti-Gastric Precancerous Lesions effects by ameliorating gastritis injury and selectively inhibiting the proliferation of gastric cancer cells rather than normal cells	Rupu He et al. (2022)
	CPW1-Se, 12.5, 25 and 50 μg/mL	Huh-7 and HepG2	vitro	Blank	CPW1-Se possessed selective anti-hepatoma activities without side effects on normal cells	Hu et al. (2022)
	The water extract of *Codonopsis lanceolata*, 2.5, 5, and 10 μg/mL	A549 cells	vitro	normal	*C. lanceolata* polyacetylenes also reduced the expression of Ras, PI3K, p-AKT, Bcl-2, cyclin D1, and CDK4 but increased the expression of Bax, GSK-3β, clv-caspase-3, and clv-caspase-9, which could be reversed by the PI3K activator 740 YP.	Wang et al. (2022)
	CPP, 10,20 and 40 μmol/L	MCF-7 cells	vitro	normal	CPP inhibits breast cancer cell proliferation and induces apoptosis through downregulation of CCHE1	Rupu He et al. (2022)
	CR, 20,40 and 60 μg/mL	H9c2 cardiomyoblast cells	vitro	None	CR could attenuate the cardiac-impaired insulin-like growth factor II receptor pathway on myocardial cells	Tsai et al. (2013)
Improve hematopoietic function and protect cardiovascular	The water extract of CR, 5 g/kg	Myocardial MIRI model	vivo	Saline	CR has significant protective effects on myocardial MIRI.	Ling (2012)
	Dangshen granule preparation 6g/(kg·d)	Establishment of heart failure mouse model by coarctation of thoracic aorta	vivo	Saline	Improve cardiac contraction function and diastolic function, increase calcium transient peak value and shorten falling time	Qun et al. (2017)
	CPPs,100、200、300 mg·kg-1	Hematopoietic stem cell senescence model	vivo	Saline	The mechanism of action may be related to the p53-p21 signaling pathway, Bax and Bcl-2 apoptosis pathway	Jiankuan Li et al. (2017)
	Alcohol extract of CR, 200 μg/mL	Human Cord Blood CD34^+^ Cells	vitro	None	CD34^+^, CD3^+^, CD19^+^ and CD71^+^ were upregulated, while the expressions of CD45^+^ and CD14^+^ were downregulated and the degree of morphological differentiation was significantly decreased	Gao (2020)
	The extract of CR, 0.5 mg/mL	A mouse embryonic stem (ES) cell-based model	vitro	ascorbic acid	The herbal extract 417W can enhance the cardiogenic differentiation of ES cells and improve the cardiac function of infarcted hearts.	Wang et al. (2021b)
	TSC, 80 mg·kg-^1^·d-^1^	Diabetic rat	vivo	Saline	Conclusion Total saponins of Codonopsis lanceolata can significantly reduce the contents of MDA, TNF-α, s ICAM-1 and s VCAM-1	Zhang et al. (2021a)
Antibacterial and antiviral effect	Alcohol extract of CR, 0.3002,0.2787,0.1450 g/mL	*Escherichia coli*, *Staphylococcus aureus*, *Streptococcus* and *Salmonella*	vitro	None	CR has antibacterial effect on *Escherichia coli*, *Staphylococcus aureus*, *Streptococcus* and *Salmonella*	Bai et al. (2015)
	Alcohol extract of CR, 0.28 g/mL	*Bacillus* catharticus, *Staphylococcus* epidermidis, *Streptococcus* hemolyticus type A and B*etc.*	vitro	None	Alcohol extract of CR has not only inhibited *Bacillus* catharticus, *Staphylococcus* epidermidis, *Streptococcus* hemolyticus type A and B, *Bacillus subtilis*, *Bacillus anthracis*, *Escherichia coli*, *Staphylococcus aureus* and *Klebsiella pneumoniae*, but also has inhibited *Bacillus anthracis* and *Salmonella typhi*	Duan et al. (2012)
	pCPPS,39 μg/mL	Duck embryonic hepatocytes (DEHs)	vitro	None	pCPPS reduces the number of DHAV, was more effective than CPPs in anti-DHAV activity	Ming et al. (2017)
Anti-aging and antioxidant effects	flavonoids from CCS, 0.25,0.5.1 mg/kg	Normal	vivo	blank	Total flavonoids of CR increased SOD activity in serum and liver, decreased MDA content, prolonged weight-bearing swimming time, increased liver glycogen and muscle glycogen storage, and decreased serum urea nitrogen level after exercise in mice	Wang et al. (2012)
	The water extract of CR, 0,3,9,15 mg/mL	*Drosophila melanogaster*	vivo	vitamin E	Water extract of Radix Codonopsis pilosula may exert anti-aging effects by regulating PI3K-Akt signaling pathway, improving the ratio of *Drosophila* probiotics to some extent, and regulating *Drosophila* phenylalanine metabolic pathway	Wu (2021)
	lobetyolin, L-tryptophan, and syringina	H_2_O_2_ induced oxidative damage model in RAW264.7 cells	vitro	blank	Anti-oxidative stress of lobetyolin, L-tryptophan, and syringin may act through total antioxidant enzymes and the Keap1-Nrf2 pathway	Liao (2022)

### 7.1 Neuroprotection

CPPs had a significant protective effect against sodium thiosulfate injury in neural stem cells (Wu et al., 2008). The study by Zhang Q et al. found that CPPs could activate PP2A to prevent AD-like tau protein hyperphosphorylation, which could rescue neuronal loss and alleviate AD-like cognitive impairment (Zhang et al., 2018). Banqiao Badix Codonopsis Polysaccharide (BCP) could effectively improve cognitive dysfunction induced by elevated GSK-3β activity in rats, and the possible mechanism was related to the downregulation of GSK-3β activity, which in turn inhibited Tau protein hyperphosphorylation and promoted neuronal development, and had some preventive or delaying effects on AD (Luo et al., 2017). Wan L et al. found that CPPs attenuate Aβ pathology in APP/PS1 mice and that downregulation of BACE1 may be a potential mechanism which could serve as a therapeutic target to attenuate cognitive deficits in AD pathology (Wan et al., 2020). Hu Y R et al. suggest that CPPs protect PC12 cells from Aβ_1-40_-induced injury, indicating that these components of CR may represent an early therapeutic option for AD patients (Hu et al., 2021). However, the therapeutic effect and mechanism of CPPs on early AD model *in vivo* and *in vitro* still need further study. CPPs improved memory consolidation impairment in mice induced by cycloheximide, and the mechanism was related to the upregulation of protein expression of CaMKII/CREB signaling pathway (Liu, 2020).

### 7.2 Protect gastrointestinal mucosa and anti-ulcer

Cui J C et al. found that CPPs had significant protective effects on four rat gastric ulcer models (stress type, anti-inflammatory pain type, acetic acid type, and pylorus ligation type). In addition, it had a significant inhibitory effect on the increase of gastric acid caused by pilocarpine, and the content of PGE_2_ in gastric juice was increased (Cui et al., 1988). Chen S F et al. observed that CR increased the content of growth inhibitors in the gastric and duodenal mucosa of rabbits, which is beneficial for the treatment of peptic ulcer (Chen S. et al., 2002). Li R L et al. discovered that the water extract of CR could reduce gastric mucosal injury and pathological damage in rats with stress ulcers and increased the content of polyamines (spermidine) in gastric mucosa. And in a subsequent study, it was found that the cell migration-promoting effect of the flavonoid-extracted part of CR was related to the influence of polyamine regulatory signaling pathways (Li et al., 2013; Li L. et al., 2014). Ma F L et al. showed that the CPPs had the effect of promoting small intestinal peristalsis in mice. Moreover, it could also promote the growth and improve the digestive ability of rats (Ma X. et al., 2014). Zhao Xiaofang studied the significant alleviating and antagonistic effects of BCP on TNBS-induced ulcerative colitis in rats, and significantly improved the damage caused by UC on colonic mucosa; The mechanism of therapeutic effect on UC may be through the activation of TLR4-NF-κB pathway to regulate the secretion of cellular inflammatory factors (IL-6, TNF-α and IL-10) and inflammatory mediators (NO), and is also associated with antioxidant and anti-inflammatory effects (Zhao, 2016). Li J K et al. first reported that inulin-type fructan CP-A may be a potential component in CR for the treatment of acute gastric ulcer. CP-A significantly increased SOD and GSH-Px activities in gastric tissues. Besides, MDA and NO content and MPO activity were decreased, and in a dose-dependent manner (*p* < 0.05) (Li J. et al., 2017). However, this experiment is only based on theory, lacking *in vivo* verification. In the future research, further animal experiments are needed to verify its anti-ulcer effect. Meng J found that CR can intervene in aging as a whole and protect the gastrointestinal tract of aging mice, OC105243318, Fam132a, RORC and 1200016E24Rik genes may be potential targets for CR to protect the aging gastrointestinal tract, and their interaction network may play an important role in CR protection of the aging gastrointestinal tract (Meng, 2021). Liu et al. revealed that TSC had a significant protective effect on colonic mucosal injury in UC rats, and the mechanism may be related to anti-lipid peroxidation, inhibition of NF-κB signaling pathway and thus regulation of inflammatory factor release (Liu et al., 2021).

### 7.3 Regulate body immunity

The methanol extract of CR has effect in regulating macrophage-mediated immune response, and promote anti-inflammatory activity (Lee et al., 2007). Lu Dangshen Oral Liquid could regulate immunosuppressed mice by promoting the development of immune organs, proliferation of immune cells and increasing the level of immune factors (Tianhui et al., 2019). CPPs enhanced the proliferation of rat splenic lymphocytes and their ability to secrete IL-2 and IL-4, and enhanced the proliferation and phagocytosis of rat macrophages and their ability to secrete TNF-α and IL-6 *in vitro*. *In vivo* animal experiments, CPPs can increase the spleen index and thymus index of immunocompromised rats, and increase the levels of IgG, IgM, IgA, C3 and C4. Its molecular mechanism may be related to the inhibition of the expression of Bax and NF-κBp-p65 proteins in immune organs, and the promotion of the expression of Bcl-2 protein in immune organs (Xu, 2018). BAI R B et al. studied that *Codonopsis pilosula* oligosaccharide (CPO), CPO could increase immune organ index, phagocytic index and immunoglobulin content, stimulate the proliferation of splenic lymphocytes (in synergy with ConA and LPS), enhance ear swelling in DTH response, promote the production of NO and cytokines (IL-2 and IFN-γ), and upregulate the expression of corresponding mRNA (Bai et al., 2020). This study is the first to show that the quality of *Codonopsis pilosula* is related to its sweet component, oligosaccharides. However, although the sugar content of CPO up to 92.7%, it is still a mixture of glycans, and its specific active ingredients need to be verified. SUN Q L et al. showed that RCAP-1 and RCAP-2 had a significant immune stimulating effect on NO production by RAW264.7 macrophages (Sun et al., 2019).

### 7.4 Anti-tumor

Codonoposide 1c is a potent apoptosis inducer that induces apoptosis in HL-60 cells (Lee et al., 2005). The saponins of *Codonopsis* could inhibit the proliferation of human hepatocellular carcinoma HepG-2 and SMMC-7721 cells. (Li, 2012; Fang et al., 2015).The water-soluble polysaccharides CPP1a and CPP1c induce apoptosis in HepG2 cells by upregulating the Bax/Bcl-2 ratio and activating caspase-3. In addition, it is cytotoxic to cervical cancer Hela cells and gastric cancer MKN45 cells. (Bai et al., 2018). Pectic polysaccharide CPP1b is significantly cytotoxic to human lung adenocarcinoma A549 cells in a dose- and time-dependent manner (Chen W. et al., 2014). More importantly, CR could exert anti-gastric precancerous effects by ameliorating gastritis damage and selectively inhibiting the proliferation of gastric cancer cells but not normal cells (He W. et al., 2022). CPW1 stabilized SeNPs are selectively tumorigenic to Huh-7 and HepG2 cells with strong anti-proliferative and pro-apoptotic activities, but do not inhibit the viability of human normal cells (Hu et al., 2022). Lobetyolin and lobetyol are the main active anti-tumor components of *Codonopsis lanceolata*, *Codonopsis lanceolata Polyacetylenes* induces apoptosis in lung cancer cells and ameliorates lung dysfunction by inactivating the Ras/PI3K/AKT pathway (Wang et al., 2022). CPPs inhibit MCF-7 cell proliferation and induce apoptosis through downregulation of CCHE1 expression (He R. et al., 2022).

### 7.5 Improve hematopoietic function and protect cardiovascular

CR attenuates insulin-like growth factor II receptor pathway damage in cardiomyocytes and reduces cardiomyocyte apoptosis (Tsai et al., 2013). CR restored LV systolic pressure (LVSP) and maximum rate of intraventricular pressure change (±dp/dtmax) to varying degrees (*Р* < 0.01), reduced LV end-diastolic pressure (LVEDP) (*Р* < 0.01), inhibited the elevation of MDA, LDH and CK, and enhanced SOD, GSH-Px, Na+, K^+^-ATP and Ca2^+^-ATP activity (Ling, 2012). CR intervention improves cardiac systolic and diastolic function to varying degrees, increases peak calcium transients and shortens fallback time (Qun et al., 2017). CPPs can retard X-ray-induced hematopoietic stem cell senescence, and its mechanism of action may be related to p53-p21 signaling pathway, Bax and Bcl-2 apoptosis pathway (Li Y. et al., 2017). The alcoholic extract of CR and potentially active compounds can promote the proliferation of hematopoietic stem cells, maintain the stemness of hematopoietic stem cells, and thus perform hematopoietic improvement functions (Gao, 2020). The extract of CR can promote the cardiogenic differentiation of embryonic stem cells and improve the cardiac function of infarcted heart (Wang J.-N. et al., 2021). *Codonopsis lanceolata* can significantly increase SOD activity, improve myocardial antioxidant level and reduce MDA expression, while significantly reducing the expression of sICAM-1, sVCAM-1 and TNF-α in rat cardiomyocytes, with certain myocardial protective effects (Zhang Y. et al., 2021).

### 7.6 Antibacterial and antiviral effect

The ethanolic extract of CR had certain antibacterial effect, while the crude polysaccharide of CR had no obvious antibacterial effect, and only had some antibacterial activity against *Salmonella* (Bai et al., 2015). In addition, it has been found that the alcoholic extract of CR has not only inhibited *Bacillus* catharticus, *Staphylococcus* epidermidis, *Streptococcus* hemolyticus type A and B, *Bacillus subtilis*, *Bacillus anthracis*, *Escherichia coli*, *Staphylococcus aureus* and *Klebsiella pneumoniae*, but also has inhibited *B. anthracis* and *Salmonella typhi* (Duan et al., 2012). Phosphorylated Codonopsis pilosula polysaccharide reduces the amount of DHAV and has stronger anti-DHAV activity than CPPs (Ming et al., 2017). In addition, studies have also found that BCP has a good preventive effect against BVDV virus (Xuan, 2019).

### 7.7 Anti-aging and antioxidant effects

It has been found that the water extract of CR can delay aging by antioxidant, but not with the activity of antioxidant enzymes SOD and CAT (Wu, 2021). Moreover, Lu-dangshen has the effect of anti-skin photoaging, and one of the possible mechanisms is that Lu-dangshen can reduce the expression of inflammatory factors in skin tissue of photoaging mice, and directly or indirectly reduce the effect of inflammatory factors and their receptors on the genes and proteins related to MAPK and PI3K signal transduction pathways (Wang, 2020). There are scholars who have used a network pharmacology approach and found that the antioxidant stress effects of lobetyolin, L-tryptophan, and syringin may act through total antioxidant enzymes and the Keap1-Nrf2 pathway (Liao, 2022). However, this experiment does not have data to support its results, which can be discussed in depth by scholars next.

### 7.8 Others

In addition to the above-mentioned pharmacological effects, CR also has a wide range of pharmacological activities in anti-stress (Hou et al., 2013), anti-inflammatory (Zhang J. et al., 2021), antifatigue (Yi et al., 2021) and antihypoxia (Yan et al., 2006; Xie et al., 2020a; Xie et al., 2020b).

In conclusion, there are many common problems in the current research on the pharmacological effects of CR, such as the lack of controlled studies between positive drugs and CR, unclear active components in related pharmacological effects and insufficient research on the mechanism of related pharmacological effects.

First of all, currently, the research on CR and its extract has a wide research prospect. But the extract is complex and its mechanism is difficult to elucidate. Therefore, it is necessary for us to further study the monomer compounds of TCM. However, up to now, studies on the pharmacological activity of CR mainly focus on CPPs, lobetyolin and TSC. The possible reason is that the other components in CR are lower and the efficacy is not obvious. Consequently, the current understanding of the active ingredients in CR is relatively limited. Therefore, scholars can use more sophisticated instrumentation methods to investigate the other components in CR.

Second, although we have made some progress in our current research on the pharmacological effects of CR, we still have a long way to go, such as the lack of research on the optimal extraction process of polysaccharide compounds in CR and the lack of research on the optimal effective dose of CR immunomodulation. Moreover, it is difficult to have an accurate indicator of clinical dosage due to the different content of polysaccharides extracted by different methods. Thus, it is necessary to establish a unified standard for the extraction of polysaccharide compounds in CR for better clinical application.

Third, Cao H F found that total saponins of *Codonopsis pilosula* nanoemulsion could enhance the regulation of cellular, humoral and non-specific immunity by TSC (Cao and Wang, 2019). In the following research, scholars may also consider developing cheaper, environmentally friendly, safe, and effective materials to replace the traditional codonopsis preparations, and developing more new dosage forms of Chinese medicine for clinical application.

Fourth, TCM has natural advantages that synthetic drugs do not have the anticancer activity of CR has been demonstrated in previous studies. But there is no doubt that the inclusion of CR in the list of anticancer drugs still requires a great deal of research. In the next step, it is necessary to establish suitable models to determine the mechanism of action, safety and dose range, as well as to perform histological and immunohistochemical assays. Besides, the antimicrobial activity of Chinese herbs, which are of wide interest because of their high efficiency, low toxicity and green. However, due to the complexity of the active ingredients of CR, the above experiments were performed only for preliminary antibacterial tests on CR extracts. Further research and studies are needed to investigate the components of the extract that have antibacterial activity and their antibacterial mechanisms.

Last but not least, Flavonoids from CCS have obvious antioxidant and anti-fatigue pharmacological effects (Wang et al., 2012). This also reminds us to strengthen research on non-traditional drug parts and local commonly used products, expand drug sources, and make rational use of drug resources. For example, the development of stems and leaves and local common products into health products or food additives can not only reduce the waste of resources but also extend the industrial chain of CR products, which has important ecological and practical significance.

In a word, we have systematically summarized the pharmacological effects of CR and provided a reference for its development and utilization for future studies. In subsequent studies, researchers can use this as a basis for in-depth studies on CR.

## 8 Toxicity

The Chinese botanical drug CR has a complex and diverse composition and can be used for both qi and fluid injuries or qi and blood deficiencies. Moreover, it is necessary for us to study the toxicity of CR in depth to provide a scientific basis for the rational clinical use of CR. In this paper, we reviewed the literature related to the toxicity of CR that we could collect so far, and the results showed that CR has no significant toxic side effects and a wide range of safety. Cheng J L et al. conducted an acute toxicity test on SPF grade KM mice with broken wall powder of CR, and no acute toxic reactions were observed in mice (Cheng et al., 2011). Scholars studied the acute toxicity of CPPs on ordinary-grade mice. The mice were oral administered with the maximum concentration of CPPs 0.5 g/mL (20 g/kg) for three times a day. There was no adverse effect on the body weight, activity, food intake, and external signs of the mice. All the mice survived in good health 7 days after administration (Feng and Gao, 2012). WU J L et al. conducted an acute toxicity test on mice with CPPs and adopted the methods of intravenous administration, intraperitoneal administration and gavage administration, respectively, and obtained the result of low toxicity of CPPs by continuous observation for 2 weeks (Wu et al., 2016); Hou L L et al. conducted a long-term toxicity test on rats with the oral solution of CPPs. No significant toxic effects were observed by analyzing hematology, blood biochemistry, weight, and organ histopathology sections of rats (Hou et al., 2016). There are also researchers conducted acute oral toxicity test, genetic toxicity test and subchronic toxicity test on ICR mice and SD rats with the extract of CR, and the results showed no significant toxicity. Moreover, no harmful effect was observed at the dose of 8.0 g/kg BW (equivalent to 16.0 g/kg BW of CR), which met the requirements of National food safety standards (Hu Z. et al., 2018). Xu X H et al. showed no significant toxic effects in the acute oral toxicity test, three genotoxicity tests (bacterial revertant mutation test, mammalian erythrocyte micronucleus test and mouse spermatocyte chromosome aberration test) and subchronic toxicity test on Wen Dang and Baitiao Dang (Xu et al., 2021; Xu et al., 2022).

In conclusion, there is no acute toxicity, subchronic toxicity, genotoxicity or long-term toxicity in CR and its extracts, and CR has a wide range of safety. Thus, CR has a high edible safety and the efficacy of tonifying the middle and replenishing qi. While worrying about “The medicine has side effects”, we cannot ignore the dual-use of some Chinese medicines as both food and medicine. Currently, CR has been included in the National List of Food and Drug Substances in Japan, Singapore and other countries. On April 24, 2018, the National Health and Wellness Commission of China issued the letter on soliciting opinions on the administration of using 9 substances such as CR as substances that are traditionally considered as both food and Chinese medicinal materials (national health office food letter [2018] No.278), clarifying the legal status of CR as food. At present, CR is not included in the list of medicinal and food products. Therefore, in the future, a large number of animal and clinical experiments should be conducted to verify its safety, so that it can be better used as a medicinal and food product for clinical treatment and healthcare, and to provide information for the development and utilization of CR in the food field.

## 9 Pharmacokinetics

Currently, there are few reports on the pharmacokinetics of CR extract and its main active components. DOU X et al. et al. demonstrated that the micronization of CR could promote the absorption and bioavailability of lobetyolin in rats (Xia et al., 2016). However, the main active ingredients of CR include CPPs besides lobetyolin. In order to clarify the advantages of ultramicro-powder more scientifically and comprehensively, it is necessary to continue to study the pharmacokinetics of CPPs *in vivo*. Dong J J et al. established the UPLC method for the determination of lobeyolin in rat plasma and found that, lobeyolin is poorly absorbed or widely metabolized in rats, and there are interactions among the components of CR extract, which leads to differences in pharmacokinetics between lobeyolin and CR extract. The reasons for the low bioavailability of lobetyolin and the methods to improve its oral absorption need to be further studied, so as to provide reference for its preparation (Dong et al., 2021). The concentration of inulin-type fructan CPA in CR was detected by fluorescent labeling method, and the *in vivo* process of CPA was analyzed. It was found that FCPA could be absorbed through blood circulation after oral administration, with low bioavailability, and its tissue distribution was wide, with long T_1/2_ and MRT (Guo et al., 2022). Scholars used UHPLC-Q/TOF-MS and UHPLC-MS/MS methods to analyze the metabolic pathways of three polyacetylene compounds from CR: lobetyol, lobetyolin, and lobetyolinin *in vivo* and *in vitro* (Xie et al., 2023)*.*

However, at present, there are few studies on pharmacokinetics of CR, and little is known about how the active ingredients of CR are absorbed from outside to inside, how they are distributed, metabolized and excreted in the body. The complexity of ingredients, uncertainty of effective ingredients and diversity of analogues of traditional Chinese medicine make it difficult to develop pharmacokinetics of traditional Chinese medicine. Therefore, we need to strengthen the work in this area, combine the exploration of pharmacokinetics with the theoretical exploration of TCM, and strengthen the study of metabolites kinetics.

## 10 Conclusion and prospect

Chinese medicine is a natural product that has advantages not found in synthetic drugs. CR, as one of the traditional Chinese medicines, can be used as both medicine and food. In this paper, the compounds extracted and separated from CR and their structural formulas were summarized systematically, which had certain reference value. Moreover, in the pharmacological section, we have summarized in detail its neuroprotective effects, protection of gastrointestinal mucosa and anti-ulcer, regulation of body immunity, anti-tumor, endocrine regulation, improvement of hematopoietic function, cardiovascular protection, anti-aging and antioxidant effects, which can provide reference for the next studies. Such an in-depth and comprehensive summary has never been seen in the previous literature on CR. In addition, in this review, we also discuss in depth the inadequacies in the existing studies on CR, and present our own views and solutions.

First, the chemical composition, especially the material basis of the medicinal effect of CR and its constitutive relationship should be studied in depth. In addition, non-traditional medicinal parts of CR, such as stems and leaves, also contain a variety of chemical components. However, relatively little research has been conducted on it. In the next step, the research on non-traditional medicinal parts of CR should be strengthened to make full use of herbal resources and avoid waste. More importantly, there are many varieties of CR. As mentioned above, the local products, *Codonopsis clematidea*, has stronger anti-free radical ability than Lu Dangshen, but it is still less exploited. Besides, in addition to traditional Chinese medicine, Dangshen is also widely used in Mongolian medicine and Tibetan doctor. For example, Weihe granule II has good effect on chronic gastritis (Li L. et al., 2014); Ganshen granules is used for deficiency of both qi and blood, dizziness, insomnia, fatigue and soreness of waist and legs (Cheng et al., 2019); ShanHuQiShiWei Pill are mainly used for cerebral thrombosis, coronary heart disease, epilepsy and various neuritis; Eighteen Flavor Dangshen Pills in the treatment of Pyocutaneous Disease, diminishing inflammation and relieving painand (Li et al., 2021). Therefore, our research on Codonopsis should not be limited to traditional Chinese medicine, but also combined with Mongolian medicine, Tibetan medicine and other clinical applications of Codonopsis, in order to facilitate a more comprehensive development and utilization of Codonopsis. In the future, we should strengthen the research on local products and establish a number of breeding bases in the concentrated distribution areas of medicinal wild resources, such as Xinjiang, Tibet and Sichuan, for artificial introduction and cultivation, so as to better develop and utilize the medicinal resources of CR.

Second, as a traditional Chinese medicine commonly used in China, CR has the characteristics of multi-component and multi-target combined effect. Currently, the indexes of quality evaluation of CR are only limited to CPPs, Atractylenolide III and lobetyolin, which still have certain shortcomings. Whether the quality evaluation of several components can represent the overall clinical efficacy of CR remains to be studied in depth. In addition, CPPs is the main active component of CR. However, polysaccharides are a series of components, and the precise isolation and preparation of polysaccharides are still difficult, the mechanism of pharmacological effect still needs to be studied in depth, and it is still challenging to obtain polysaccharide components that can represent the characteristics of CR. We need to establish scientific and reasonable quality control standards to contribute to the safe and rational use of CR. In the future, scholars can use low-cost and environmentally friendly UPLC-QDA and UPC^2^ methods to screen quality markers by spectral-effect relationships or network pharmacology.

Thirdly, most of the processing methods of CR have been extended to the ancient methods without specific process parameters, which is not conducive to mass industrial production. In addition, the different processing methods had different effects on the content of the components in CR. Therefore, in the next study, scholars should focus on establishing a unified and scientific process standard, quantifying and specifying the processing ingredients, temperature, time and other factors that he would affect the quality of the product of CR.

Fourthly, CR is a natural medicine that can be used as both food and medicine, with low toxicity and high edibility. However, CR is not listed in the table of contents of homology of medicine and food in China. In subsequent studies, it is necessary to establish more animal and clinical trials to verify its safety. In addition, researchers should strive to develop more edible products using CR so that it can be better used as a health product in the daily life of people.

In conclusion, this paper reviews the research results on the botany, ethnopharmacology, phytochemistry, analysis method and quality control, processing methods, pharmacological effects, pharmacokinetics and toxicity of CR in recent years, and puts forward some opinions and suggestions to provide new ideas for further development of the plant resources of CR.
