# Supplementary material for: A review of the botany, ethnopharmacology, phytochemistry, analysis method and quality control, processing methods, pharmacological effects, pharmacokinetics and toxicity of codonopsis radix
Source: Front Pharmacol. 2023 Apr 6;14:1162036. doi: 10.3389/fphar.2023.1162036 (PMC10117688; doi:10.3389/fphar.2023.1162036)
Supplement: Supplementary file 1 [file DataSheet1.docx]

Supplementary Material

**A review of the botany, ethnopharmacology, phytochemistry, analysis method and quality control, processing methods, pharmacological effects, pharmacokinetics and toxicity of Codonopsis Radix**

**Jiaojiao Dong 1, Yexin Na 1, AJiao Hou 1, Shihao Zhang 1, Huan Yu 1, Senwang Zheng 1, Wei Lan 2*, Liu Yang1***

# * Correspondence:

# Wei Lan: College of Traditional Chinese Medicine, Xinjiang Medical University, Shangde North Road, Shuimogou District, Urumqi, Xinjiang 830017, China. Email: lanwei516@sina.com

# Liu Yang: College of Pharmacy, Heilongjiang University of Chinese Medicine, No. 24 Heping Road, Xiangfang District, Harbin 150040, China. Email: hxk_yl@163.com.Supplementary Figures and Tables

## Supplementary Tables

**Supplementary Table 1:** The sources and characteristics of the confusions of CR.

| **NO.** | **Chinese name** | **Latin name** | **Properties and characteristics** | **Distribution Area** | **Effect** | **Ref.** |
| --- | --- | --- | --- | --- | --- | --- |
| 1. | Yinchaihu | Stellaria dichotoma L. | It is cylindrical-like, with light brown to light brown surface, hard and brittle texture, easy to break, uneven section, cracks, thin skin, and yellow and white radial texture on the wood; The smell is small and sweet. | Distribution in Mongolia, Russia and China; China is distributed in Inner Mongolia, Liaoning (Chifeng), Shaanxi, Gansu and Ningxia. | Clearing deficiency heat | Chinese Pharmacopoeia,2020 edition |
| 2. | Miguoqin | Sphallerocarpus gracilis | The cylindrical surface of rhizome is brown with longitudinal fine texture and the cross section of transverse link is yellow, delicate and hollow Slightly fragrant and slightly numb tongue | Produced in Heilongjiang, Jilin, Liaoning, Hebei, Shanxi, Inner Mongolia, Gansu, Xinjiang, Qinghai and other places | Dispel kidney cold and treating skin purulence. | (Pi et al., 2013) |
| 3. | Fangfeng | Saposhnikovia divaricata (Turcz.) Schischk. | It is a long cone or a long cylinder, the lower part is tapered, some are slightly curved, the surface is grayish brown or tan, the root head has obviously dense ring patterns, the cross section is uneven, the skin is tan to brown, there are cracks, and the wood is yellow. Special flavor, slightly sweet taste. | Northeast China, North China, Shaanxi, Gansu, Ningxia, Shandong and other places. | Dispelling exterior syndrome, eliminating dampness, relieving pain and spasmolysis. | Chinese Pharmacopoeia,2020 edition |
| 4. | Jinqianbao | Campanumoea javanica Bl. | It is cylindrical, with a short rhizome at the head and branches at the lower part; The surface is grayish yellow, with irregular longitudinal wrinkles and many pimples; Hard, easily broken, uneven section, white or yellowish white; It is mild, slightly sweet and chewy. | Guizhou, Sichuan, Yunnan, Guangdong, Guangxi, etc. | Invigorating qi, stopping bleeding and promoting lactation. | (Yang et al., 2015) |
| 5. | Shishengyingzicao | Silene tatarinowii Regel | There is no "lion's head" | Produced in Hebei, Inner Mongolia, Shanxi, Henan, Hubei, Hunan, Shaanxi, Gansu, Ningxia, Sichuan (East) and Guizhou. | Clearing heat and cooling blood, tonifying deficiency and tranquilizing mind. | (Yao, 2013) |
| 5. | Guanhuadangshen | Codonopsis tubulosa Kom. | The appearance is similar to that of Codonopsis pilosula, but there are few or no annular horizontal stripes; All of them have most irregular longitudinal grooves and edges, which are firm in quality, white in color, slight in breath, slightly sweet in taste, and chewy. | Produced in western Guizhou (Nayong and Panxian), southwestern Sichuan and Yunnan (Mengzi, Dali and Lanping). It is also distributed in the north of Myanmar. | It has the function of nourishing the middle and invigorating the qi, strengthening the spleen and benefiting the lung. | (Sun et al., 2007) |

## Supplementary Figures

|  |
| --- |
|  |

**Supplementary Figure 1.** The structure of alkaloids and nitrogenous compounds of CR.

| 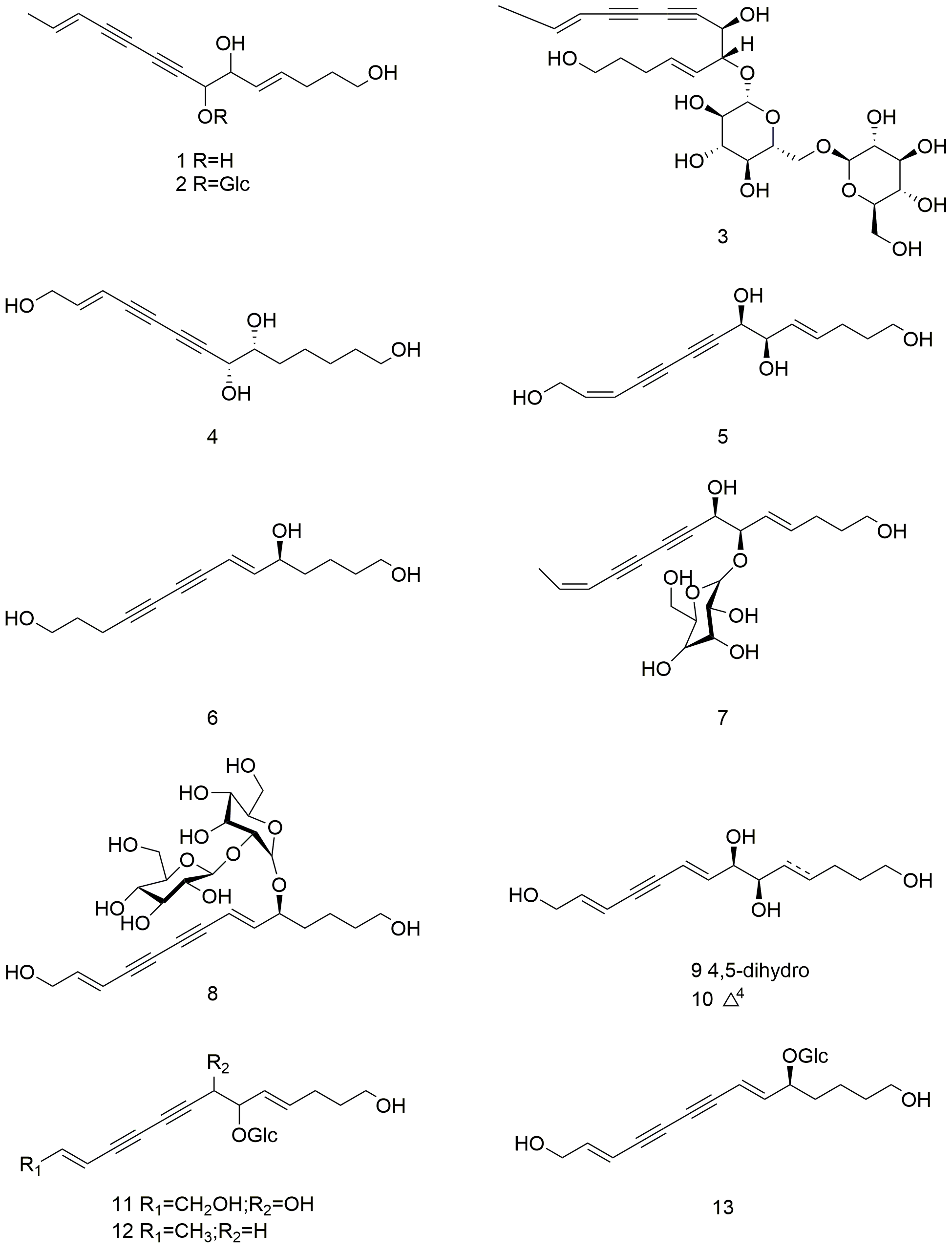 |
| --- |
| 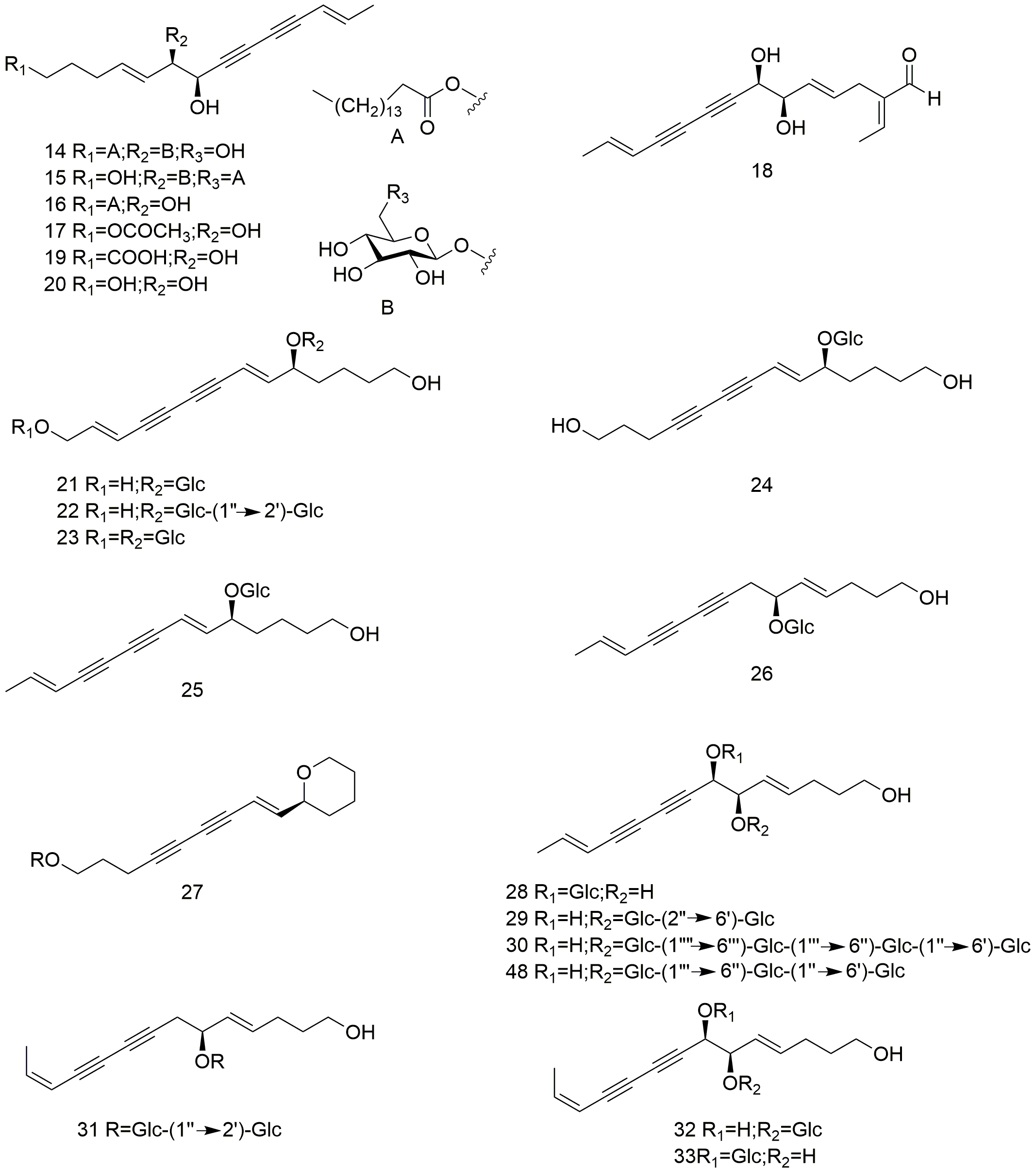 |
| 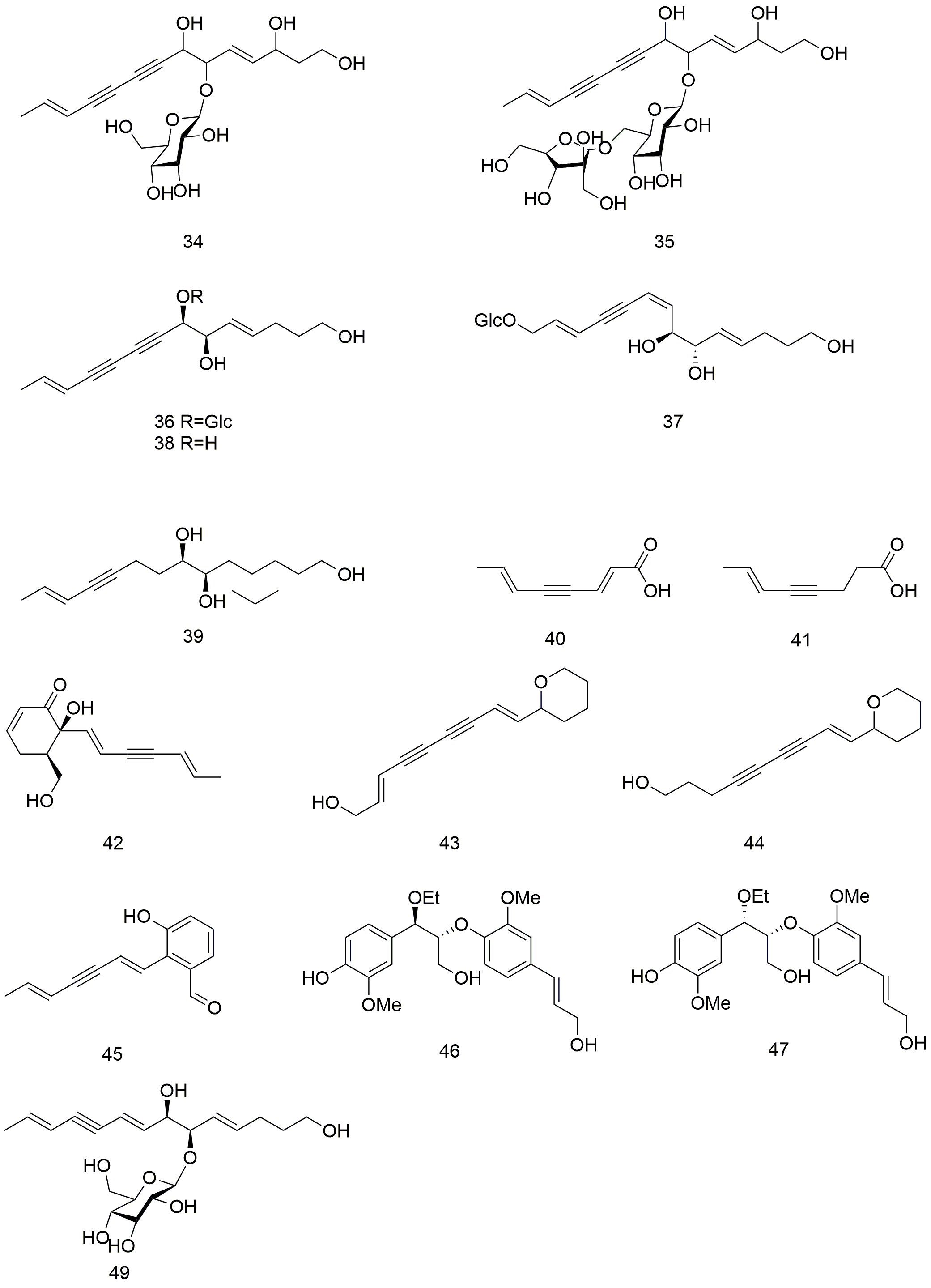 |

**Supplementary Figure 2.** The structure of Alkynes, Polyacetylenes and their glycosides compounds of CR.

| 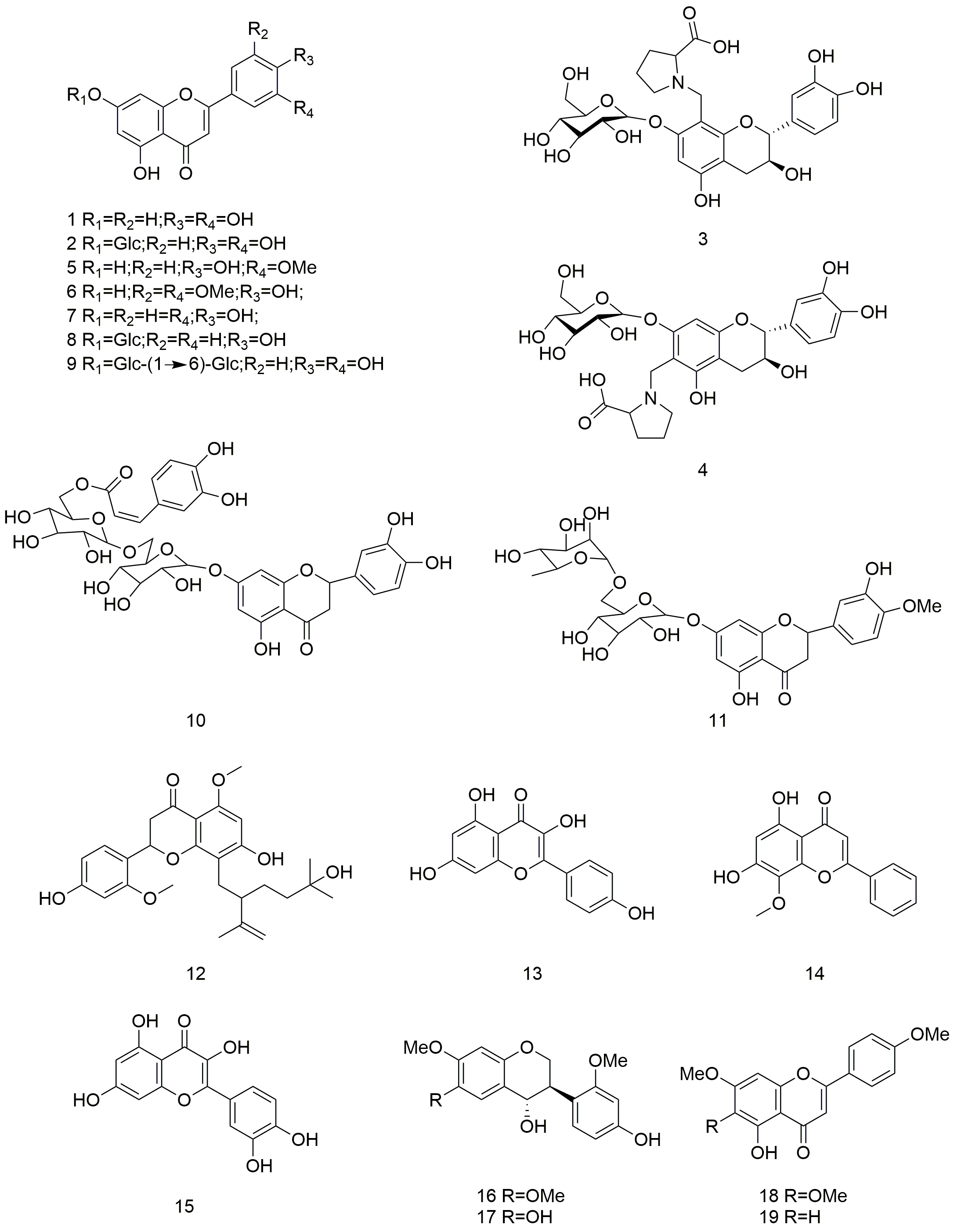 |
| --- |
| 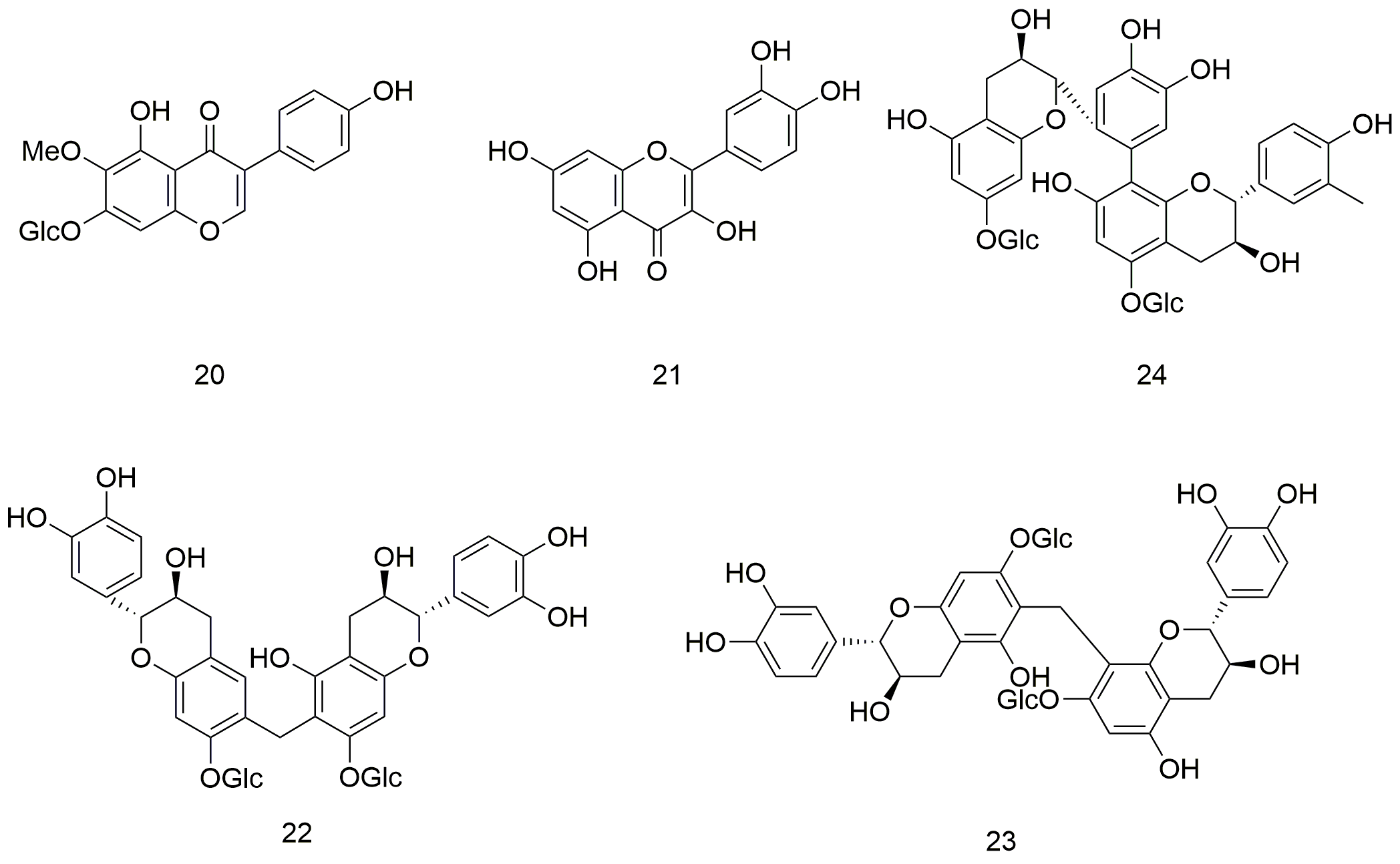 |

**Supplementary Figure 3:** The structures of flavonoids compounds of CR.

| \| 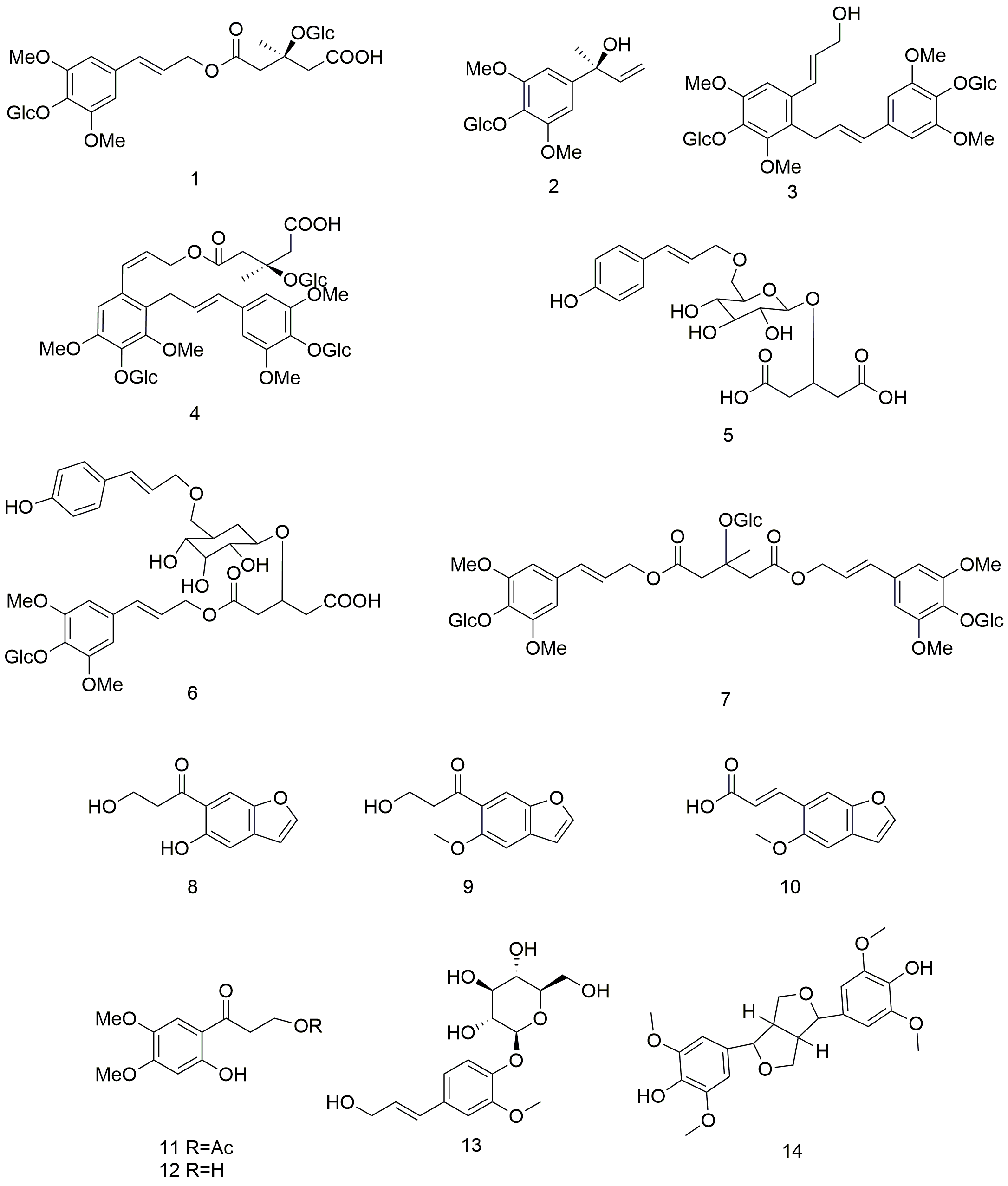 \| \| --- \| \| 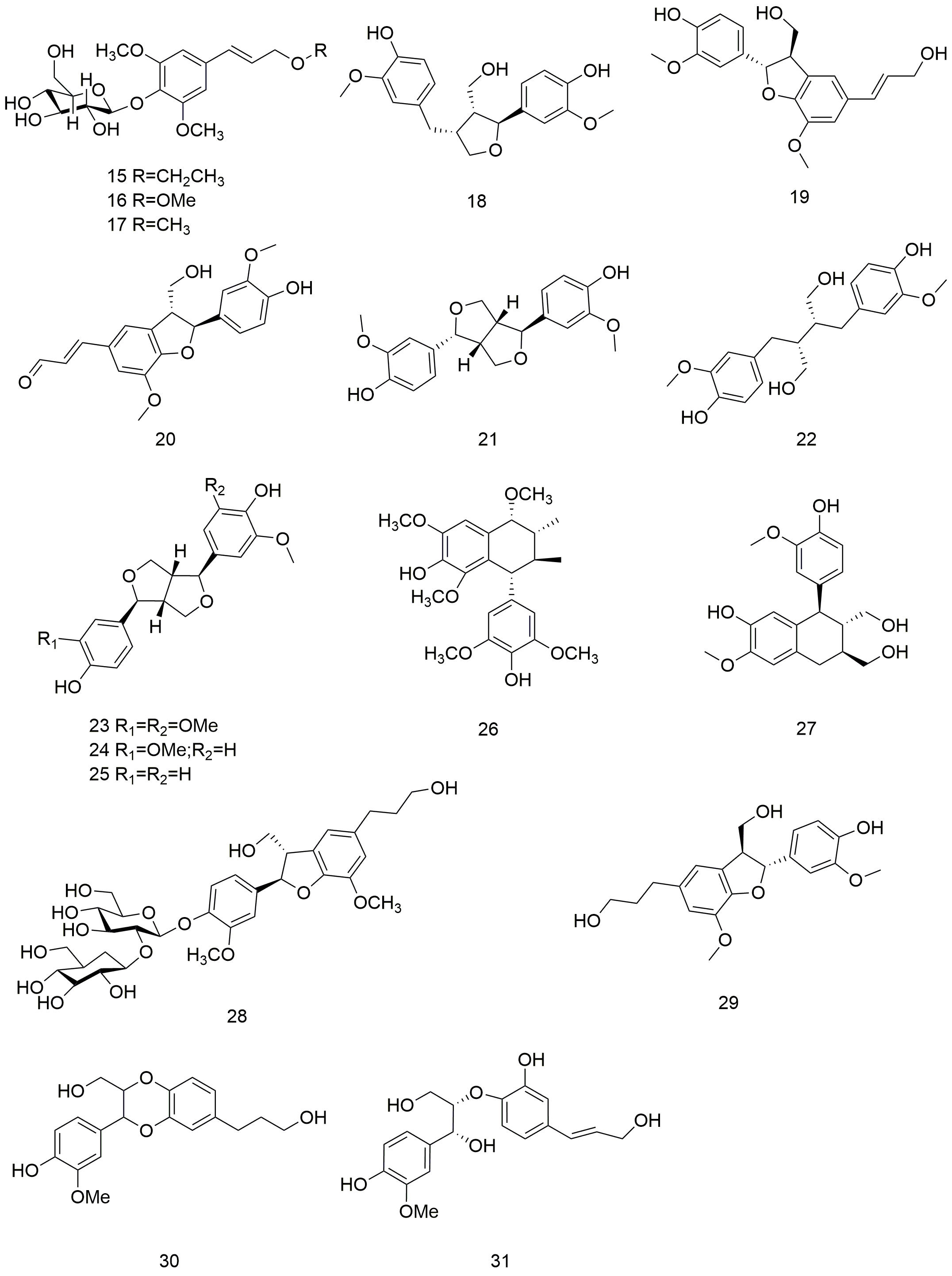 \| |
| --- | --- | --- |
|  |

**Supplementary Figure 4**. The structure of lignans and their glycosides compounds of CR.


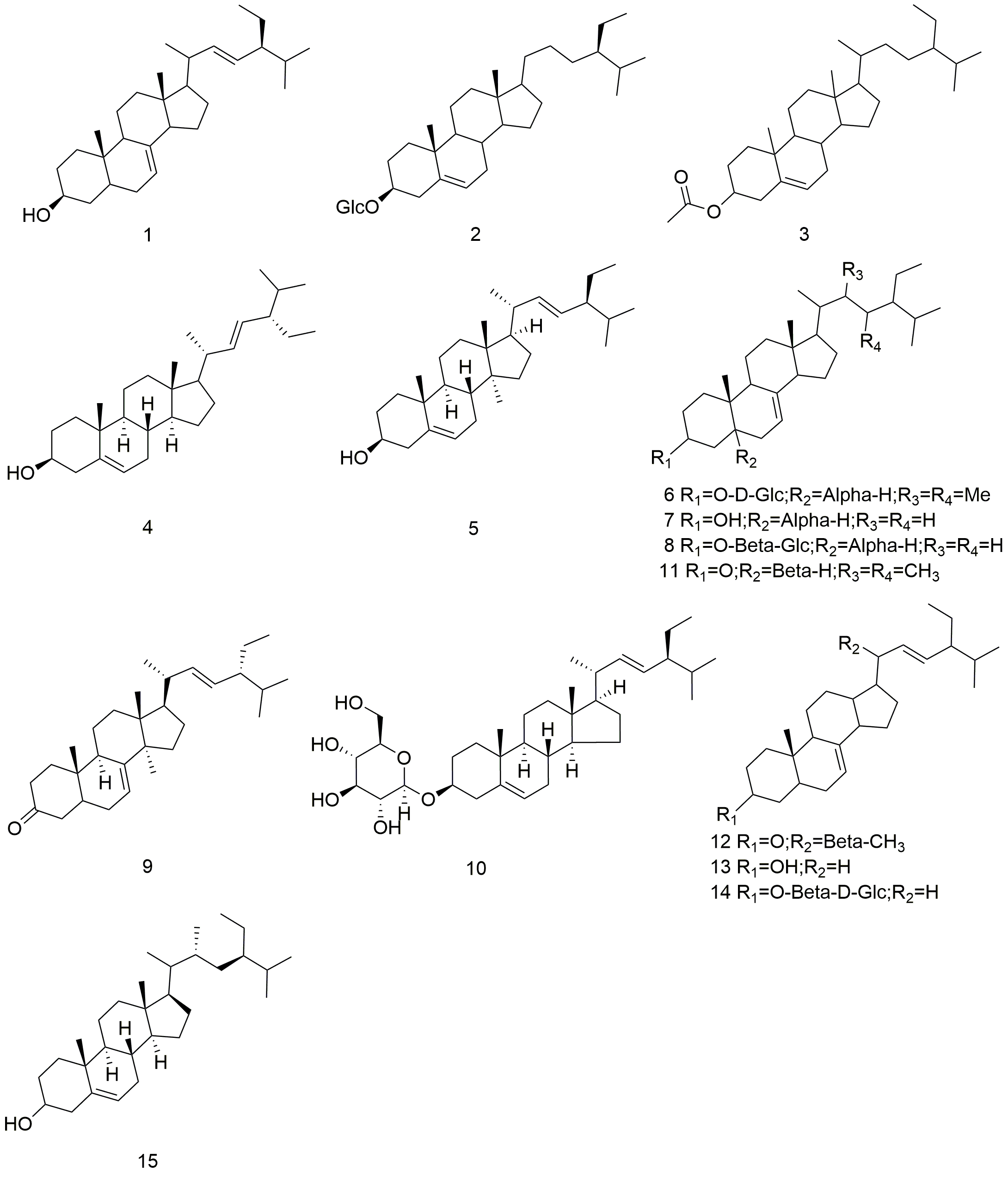


**Supplementary Figure5.** The structure of steroids and their glycosides compounds of CR.

| 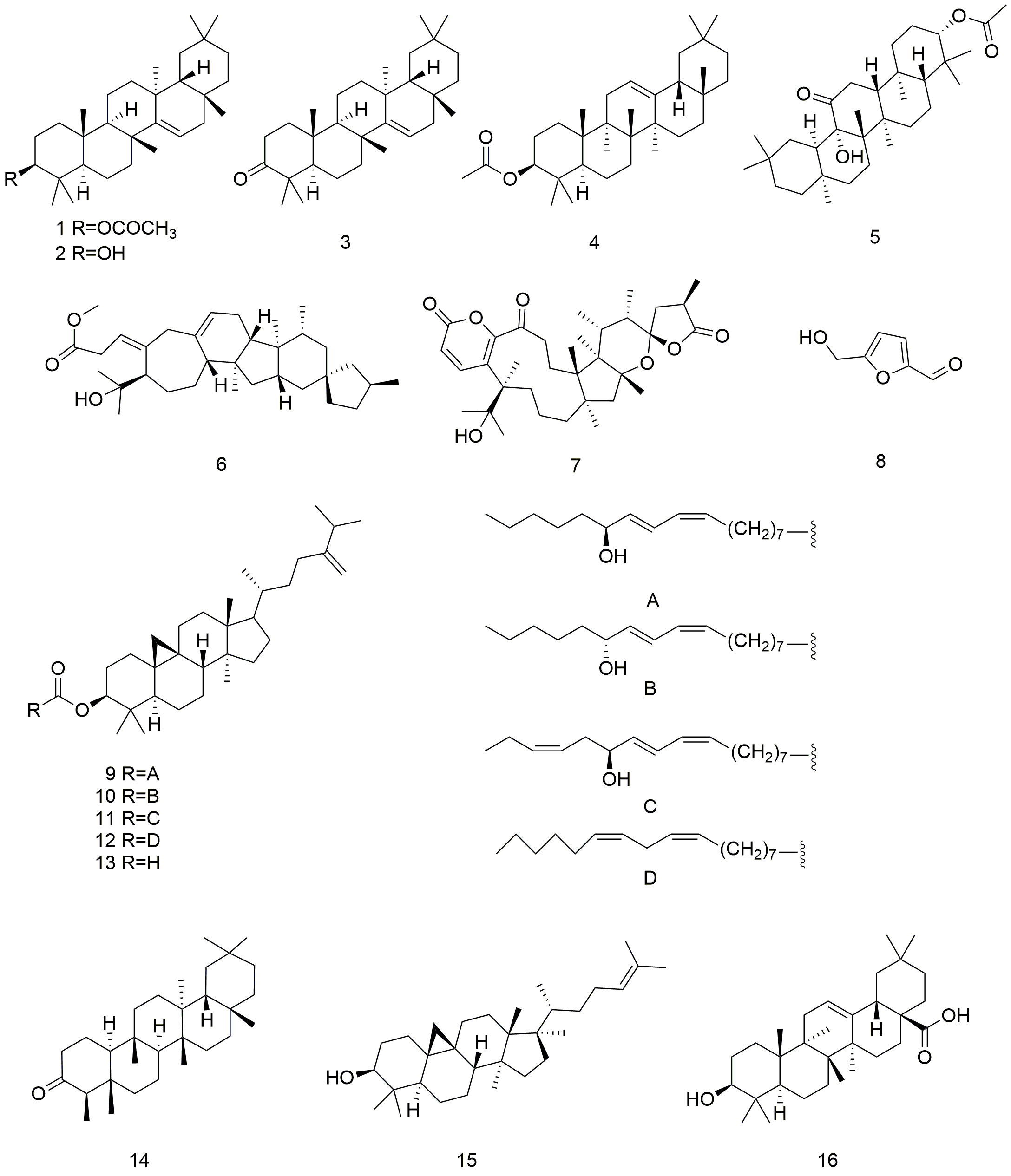 |
| --- |
| 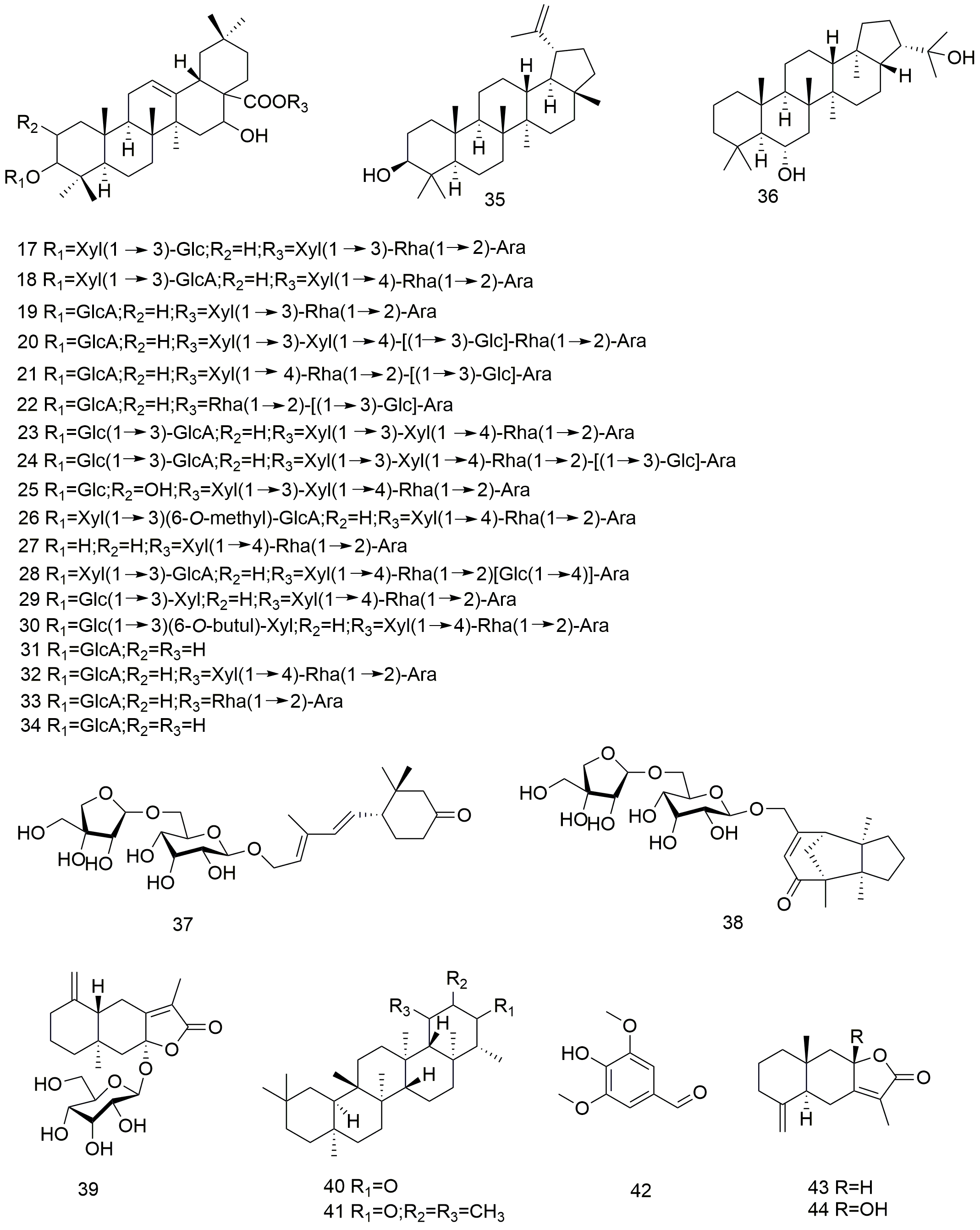 |
| 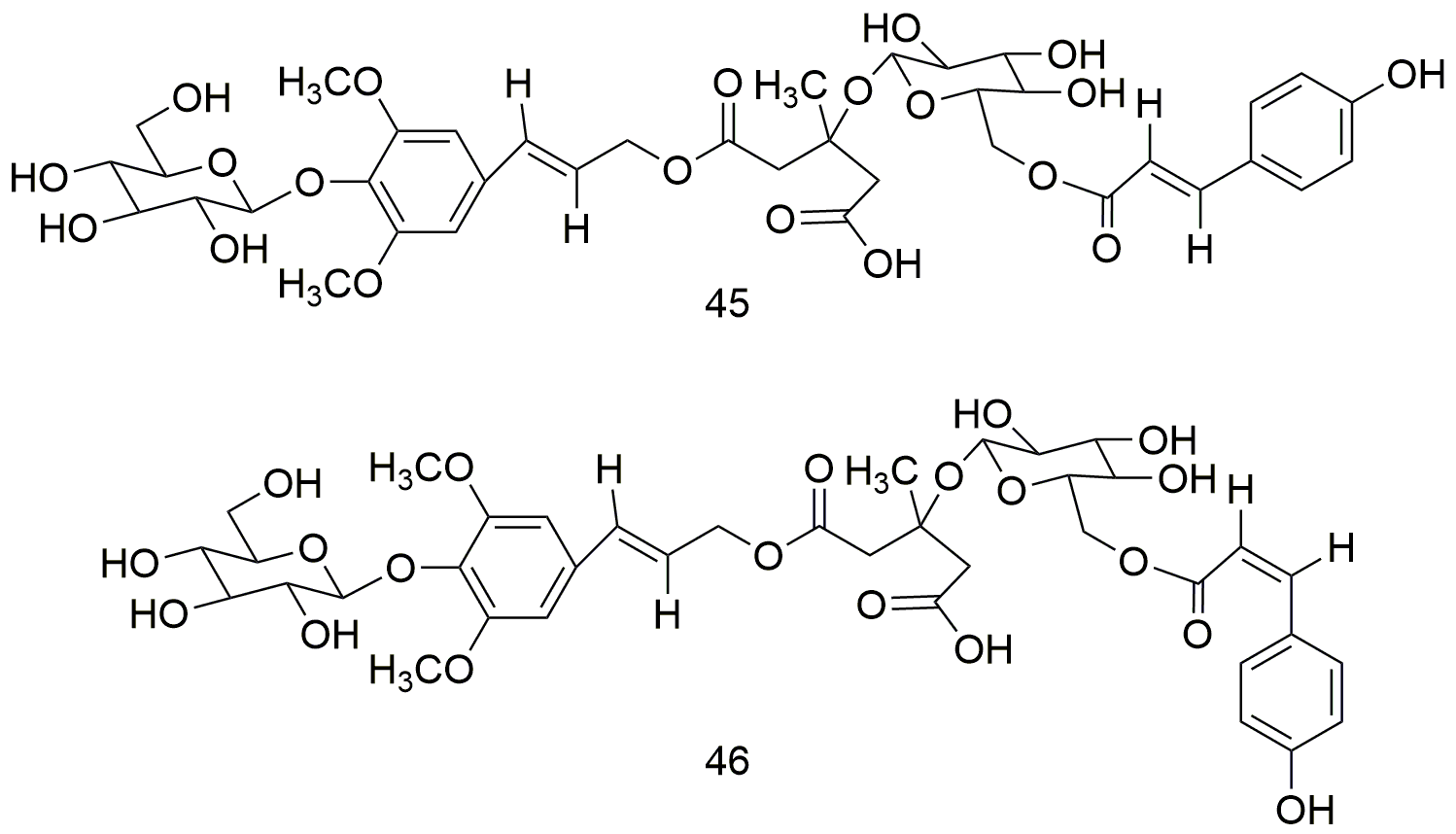 |

**Supplementary Figure6.** The structure of terpenes compounds of CR.

| \|  \| \| --- \| \|  \| |
| --- | --- | --- |
|  |

**Supplementary Figure7.** The structure of organic acids compounds of CR.


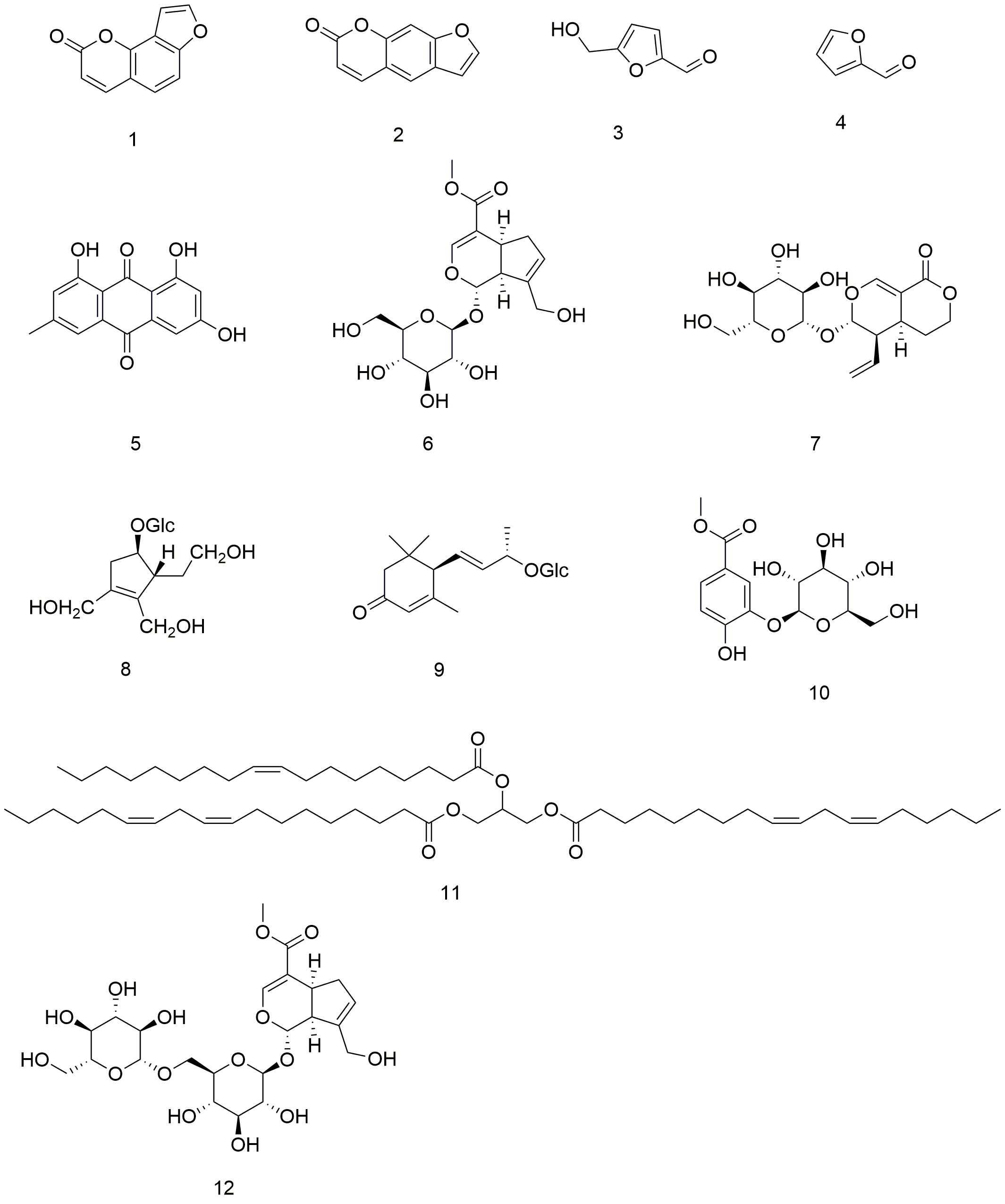


**Supplementary Figure8.**Other components of CR.
